# Gene‐based and pathway‐based testing for rare‐variant association in affected sib pairs

**DOI:** 10.1002/gepi.22291

**Published:** 2020-04-01

**Authors:** Razvan G. Romanescu, Jessica Green, Irene L. Andrulis, Shelley B. Bull

**Affiliations:** ^1^ Lunenfeld‐Tanenbaum Research Institute Sinai Health System Toronto Ontario Canada; ^2^ Centre for Healthcare Innovation, Rady Faculty of Health Science University of Manitoba Winnipeg Manitoba Canada; ^3^ Department of Molecular Genetics University of Toronto Toronto Ontario Canada; ^4^ Division of Biostatistics, Dalla Lana School of Public Health University of Toronto Toronto Ontario Canada

**Keywords:** burden tests, familial tests, pathway testing, rare variant tests, sib‐pair testing

## Abstract

Next generation sequencing technologies have made it possible to investigate the role of rare variants (RVs) in disease etiology. Because RVs associated with disease susceptibility tend to be enriched in families with affected individuals, study designs based on affected sib pairs (ASP) can be more powerful than case–control studies. We construct tests of RV‐set association in ASPs for single genomic regions as well as for multiple regions. Single‐region tests can efficiently detect a gene region harboring susceptibility variants, while multiple‐region extensions are meant to capture signals dispersed across a biological pathway, potentially as a result of locus heterogeneity. Within ascertained ASPs, the test statistics contrast the frequencies of duplicate rare alleles (usually appearing on a shared haplotype) against frequencies of a single rare allele copy (appearing on a nonshared haplotype); we call these allelic parity tests. Incorporation of minor allele frequency estimates from reference populations can markedly improve test efficiency. Under various genetic penetrance models, application of the tests in simulated ASP data sets demonstrates good type I error properties as well as power gains over approaches that regress ASP rare allele counts on sharing state, especially in small samples. We discuss robustness of the allelic parity methods to the presence of genetic linkage, misspecification of reference population allele frequencies, sequencing error and de novo mutations, and population stratification. As proof of principle, we apply single‐ and multiple‐region tests in a motivating study data set consisting of whole exome sequencing of sisters ascertained with early onset breast cancer.

## 1 INTRODUCTION

Literature on methods for genetic association analysis of rare variants under a case–control design is extensive, but relatively few methods exist to test for association under an affected sibling pair design (Chen, Weinberg, & Chen, 2016; Epstein et al., 2015; Gong et al., 2019; Guo & Zhou, 2019; K. H. Lin & Zöllner, 2015). This represents a significant gap because tests involving sib pairs have been shown to be more powerful than testing an equivalent number of cases and controls (Epstein et al., 2015; Sha & Zhang, 2015; Teng & Risch, 1999; Zöllner, 2012). From a design perspective, comparisons using siblings provide a natural way to control for many potentially confounding covariates, both genetic and environmental.

Tests for association of rare variants (RVs) with binary traits using affected sib pairs (ASPs) treat the count of RV alleles as the outcome variable. The idea developed by Epstein et al. (2015) is that rare susceptibility alleles will appear more frequently on haplotypes shared identical by descent (IBD), compared to those not shared IBD. Thus, regressing the rare allele count in a region on the corresponding IBD information for that sib pair is one way of testing for association within the region. While this approach to analyzing the ASP design is shown to have good properties in reasonably large samples, our investigations of relationships between rare allele counts and haplotype sharing have led us to alternate, more refined test statistics. Rare alleles appearing in duplicate in a sib pair will very likely be shared IBD; single rare alleles, that is appearing only once, will certainly be nonshared. Similar reasoning to that above suggests that duplicate alleles should be enriched in susceptibility regions. In this report we demonstrate through extensive simulation studies that this alternative counting method leads to more powerful tests of association than regression on IBD. We develop two tests at the region level, and extend them to test at the pathway level. Overall, the aim of our approach is to increase power to detect weaker signals, such as medium to low penetrance variants clustered in a region; or very rare, family‐specific mutations that operate through a shared disease mechanism (a pathway).

## 2 METHODS

Assume we are testing a genomic region which has been filtered on minor allele frequency (MAF) information from population reference panels (e.g., 1000 Genomes, Exome Sequencing Project [ESP 6500], UK Biobank). This produces *j* = 1,2, …, *R* loci with rare alleles (e.g., defined as MAF < 0.1%). For a study of *N* families each with two affected siblings, define $Q_{\mathrm{ij}}$ to be the number of copies of the rare allele at locus *j* for sibpair *i*, so that $Q_{\mathrm{ij}}\in \{0,\text{…},4\}$; and define $Q_{i\cdot }=\sum_{j=1,\text{…},R_{\;}}Q_{ij}$. Also, let Z_*ij*_ denote the number of alleles shared IBD for sibpair *i* at locus *j* $\left(Z_{ij}=0,1,2\right)$. We assume no recombination within a region, and for ease of notation, drop the subscript *j* from *Z*_*ij*_ unless otherwise specified. Although the method we develop specifies families with two affected siblings, the analysis can accommodate families with more affected sibs, by including all pairs of siblings as separate ASPs. For application to datasets with many large sibships, valid variance estimation might entail an adjustment for familial correlation.

Initially, we are interested in testing for a signal in a single, contiguous genetic region. This case is most commonly assumed in the RV association literature, and often corresponds to testing at the gene level (Derkach, Lawless, & Sun, 2014; S. Lee, Abecasis, Boehnke, & Lin, 2014; Wu et al., 2011, and others). Gene‐level testing reduces the multiple testing burden of marginal testing at each SNP. This benefit can be further extended if multiple genetic signals are captured in a pathway, that is a collection of genetic regions related by biological role or function; we discuss this subsequently.

### 2.1 Epstein's test

Epstein et al. (2015) model the dependence of $Q_{i∙}$ on $Z_{i}$, as summarized (in our notation) via the following regression equations:

$$E\left[Q_{i\cdot }|Z_{i}\right]=4\mu_{0}+2\left(\mu_{1}-\mu_{0}\right)Z_{i}$$

$$Var\left[Q_{i\cdot }|Z_{i}\right]=4\sigma_{0}^{2}+2Z_{i}\left(2\sigma_{1}^{2}-\sigma_{0}^{2}\right),$$

which assume that rare allele counts have a different mean $\left(\mu_{0},\mu_{1}\right)$ and variance $\left(\sigma_{0}^{2},\sigma_{1}^{2}\right)\;$ depending on whether the haplotype they come from is shared IBD or not. To test if a region is associated with disease susceptibility, that is $(\mu_{1}-\mu_{0})>0$ in that region, they first estimate $\sigma_{0}^{2},\;\sigma_{1}^{2}$ from sibpair data, and use them as weights to compute a test statistic

$$Y_{\mathrm{burden}}=\frac{U}{\sqrt{V}},$$

where U and V are based on weighted sums of $Q_{i∙}$'s. Then $Y_{\mathrm{burden}}$ is asymptotically standard normal under the null hypothesis of ${(\mu }_{1}-\mu_{0})=0$. A brief summary of the derivation is provided in Appendix A.

### 2.2 Allelic parity test

At the sib‐pair level, we define $S_{i}=\sum_{j=1,\text{…},R}I\left\{Q_{ij}=1\right\}$ and $D_{i}=\sum_{j=1,\text{…},R}I\left\{Q_{ij}=2\right\}$, i=1,…,N, which sum rare allele counts across a haplotype for single copy and duplicate variants, respectively. Here, we let *μ*_*j*_ be the frequency of the rare alleles at locus j (j=1,…,R) in the source population (assumed known, for now). Further, denote $\mu =\sum \mu_{j}$. We express the means and variances of $S_{i}$ and $D_{i}$ in terms of the $\mu_{j}$, conditional on haplotype sharing, under the null hypothesis that there are no susceptibility variants in the testing region. These derivations are presented in Appendix B, and results are summarized in Table 1; here, $\tau_{l}^{D},\tau_{l}^{S},\;l=0,1,2$ denote the conditional means of $D_{i}$ and $S_{i}$, given $Z_{i}=l$ (i.e., $\tau_{0}^{D}=E\left[D_{i}|Z_{i}=0\right]$, etc.). Parameter k, which will be estimated from study sample data, is introduced to account for within‐region linkage disequilibrium (LD) in the variance computation and acts as an overdispersion factor, that is arising from positive correlations between RVs within the region.

**Table 1:** Means and variances for counts $S_{i}$ and $D_{i}$ conditional on identical by descent sharing ($Z_{i}$)

*Z*_*i*_	$\tau_{Z_{i}}^{D}$	$Var(D_{i}|Z_{i})$	$\tau_{Z_{i}}^{S}$	$Var(S_{i}|Z_{i})$
0	$\sum 6\mu_{j}^{2}$	$k\sum^{\;}6\mu_{j}^{2}$	$\sum \;4\mu_{j}(1-3\mu_{j})$	$k\sum 4\mu_{j}(1-7\mu_{j})$
1	$\sum \mu_{j}(1-\mu_{j})$	$k\sum \mu_{j}(1-2\mu_{j})$	$\sum 2\mu_{j}(1-2\mu_{j})$	$k\sum 2\mu_{j}(1-4\mu_{j})$
2	$\sum 2\mu_{j}(1-\mu_{j})$	$k\sum 2\mu_{j}(1-3\mu_{j})$	0	0

*Note*: Expressions are accurate to second order in $\mu_{j}$.

Under the null we expect no systematic differences between the MAFs in affected versus source populations; we write $\left(\mu_{\cdot }^{aff}-\mu_{\cdot }\right)=0,$ where $\mu_{\cdot }^{aff}=\sum_{j=1,\text{…},R}\mu_{j}^{aff}$ and $\mu_{j}^{aff}$ is the frequency of the rare allele at locus *j* in the affected population. Under the alternative of some variants in the region being penetrant, the ascertainment of the study sample will be reflected in a higher count of rare alleles in the region, that is $\left(\mu_{\cdot }^{aff}-\mu_{\cdot }\right)>0$. Although an exact quantification of such increase will depend on the genetic model—which is assumed unknown—it is nevertheless possible to make qualitative observations. In particular, while we expect an enrichment in both single and duplicate counts, the frequency of duplicate alleles will increase proportionately more than the frequency of single alleles. This occurs because siblings that share a susceptibility allele are more likely to be both affected, and hence ascertained into the study, compared to pairs where one sib is an affected carrier and the other is an environmental case, or where siblings carry different susceptibility alleles. Table 2 illustrates that the increase in D from the null to the alternative is greater than the increase in *S*. The numbers in each cell are expected sums of $S_{i}$'s and $D_{i}$'s over the entire sample under the null, stratified by IBD state. These are obtained by multiplying the means in Table 1 by the expected number of samples in each $Z_{i}$ category, that is by $N\times P(Z_{i})$, where $P(Z_{i})=\left(\frac{1}{4},\frac{1}{2},\frac{1}{4}\right)$ for $Z_{i}=(0,1,2)$. The shading in each cell signifies the expected increase in that count under the alternative compared to the null (darker shading means a higher proportional increase). With this setup, a test statistic for evidence of association has the general form

$$T=\sum_{l=0}^{2}c_{l}^{D}\sum_{\left\{i:Z_{i}=l\right\}}(D_{i}-\tau_{l}^{D})+\sum_{l=0}^{2}c_{l}^{S}\sum_{\left\{i:Z_{i}=l\right\}}\left(S_{i}-\tau_{l}^{S}\right),$$

where $c_{l}^{D},c_{l}^{S},\;l=0,1,2$ are contrast weights in the comparison of the different $Z_{i}$ strata. As discussed above, one version of this test statistic contrasts the columns of Table 2, that is $2\sum_{i=1,\text{…},N}D_{i}-\sum_{i=1,\text{…},N}S_{i}$, which has a mean of zero at first order of *μ*_*j*_, assuming no RV association and no excess IBD sharing (Appendix B). Standardizing this expression leads to

$$T_{ap}=\frac{2\bar{D}-\bar{S}-6\sum_{j}\mu_{j}^{2}}{\;\sqrt{\frac{3}{N}\hat{k}\left[\sum_{j}\mu_{j}\left(2-\mu_{j}\right)\right]}}∼t\left(df=2N-2\right),$$

under the null. We call this the allelic parity statistic, because it is based on the parity relation for RVs under the null that expected counts of duplicates are half the counts for singles. The overdispersion k is estimated from data as

$$\hat{k}=\frac{s_{S}^{2}+s_{D}^{2}}{3\sum_{j=1,\text{…},R}\mu_{j}\left(1-\;\frac{19}{6}\mu_{j}\right)},$$

where $s_{S}^{2}$ and $s_{D}^{2}$ are the sample variances of $S_{i}$ and $D_{i}$, which reflect covariances among the RV loci, and provide robustness to within‐region LD. Appendix B provides detailed derivations.

**Table 2:** Expected total counts of single and duplicate alleles in the sample, stratified by $Z_{i}$, under the null

IBD sharing	Contribution to $\sum D_{i}$	Contribution to $\sum S_{i}$
$Z_{i}=0$	0	*Nμ*.
$Z_{i}=1$	***Nμ*./2**	*Nμ*.
$Z_{i}=2$	***Nμ*./2**	0
Total	***Nμ*.**	2*Nμ*.

*Note*: Bold indicates that a higher magnitude of proportional increase is expected under the alternative. Here, $\mu_{∙}=\sum_{j=1,\text{…},R}\mu_{j}$, and expressions are accurate to first order.

The $\mu_{j}$ parameter is the MAF of the variant at locus j, which we assume to be known. Values can be determined from external reference population panels, and ideally the genomic panel closely matches the genetic characteristics of the source population for the ascertained ASPs. If, however, the general level of enrichment in all RVs across the genome is consistently and substantially elevated in the sample compared to reference panels, then a genome‐wide correction may be necessary to account for systematic differences; we discuss what such a correction might be when we consider robustness to misspecification of MAFs.

We also formulate a version of $T_{\mathrm{ap}}$ that is self‐contained, in that it does not use externally supplied $\mu_{j}$. This follows from estimating the sum of $\mu_{j}$ empirically by $\frac{Q_{∙∙}}{4N}$, and dropping $O\left(\mu_{j}^{2}\right)$ terms (shown in Appendix C). Thus, this version of the allelic parity test (which we call empirical) is

$$T_{\mathrm{ap}-\mathrm{emp}}=\frac{2\bar{D}-\bar{S}}{\sqrt{\frac{1}{N}\left(s_{S}^{2}+s_{D}^{2}\right)\left(2+\frac{4Q_{∙∙}}{3N}\right)}}∼t\left(\mathrm{df}=2N-2\right).$$

### 2.3 Weighted allelic parity test

Based on Table 2, it is possible to distill a more powerful test by contrasting only the strongest signal, that is the $D_{i}$ for $Z_{i}=1$ or 2, with the corresponding mean under the null of no RV association. Because the variances of $D_{i}$ in the two strata are different, to increase efficiency we apply inverse variance weights to the contribution of the strata (i.e., by the inverse standard deviation of $D_{i}$ given $Z_{i}$). The test statistic we obtain is

$$\begin{array}{ll} T_{\mathrm{ap}-w}= & \frac{1}{\sqrt{\hat{k}\left(n_{Z_{i}=1}+n_{Z_{i}=2}\right)}}(\sum_{\left\{i:Z_{i}=1\right\}}\frac{D_{i}-\sum \mu_{j}\left(1-\mu_{j}\right)}{\sqrt{\Sigma \mu_{j}\left(1-2\mu_{j}\right)}}\\ & +\sum_{\left\{i:Z_{i}=2\right\}}\frac{D_{i}-\Sigma 2\mu_{j}\left(1-\mu_{j}\right)}{\sqrt{2\Sigma \mu_{j}\left(1-3\mu_{j}\right)}})∼t\left(2N-2\right), \end{array}$$

where $n_{Z_{i}=1,2}$ stands for the number of sib pairs with $Z_{i}=1$ and 2, and $\hat{k}$ is computed as above.

### 2.4 Pathway extensions

In the multiple region case, assume we have a collection of *p* different genetic regions (e.g., comprising a pathway), and each of these has $R_{q}$ rare variants after filtering, q=1,2,…,p. Quantities $S_{i}$ and $D_{i}$ are defined in a similar way as above, but are now specific to a genomic region denoted by an extra subscript q,q=1,…,p, that is $S_{\mathrm{qi}}$ and $D_{\mathrm{qi}}$. Also let $S_{i}^{\pi }=\sum_{q=1}^{p}S_{\mathrm{qi}}$, and $D_{i}^{\pi }=\sum_{q=1}^{p}D_{\mathrm{qi}}$, that is the sums of these quantities across the entire pathway, for one sib pair. The multiple region allelic parity test statistic has a similar form as in the single region case, namely

$$T_{\mathrm{ap}}=\frac{2\bar{D^{\pi }}-\bar{S^{\pi }}-\sum_{q=1}^{p}\sum_{j=1}^{R_{q}}6\mu_{\mathrm{jq}}^{2}}{\sqrt{\frac{1}{N}\hat{k}\sum_{q=1}^{p}\sum_{j=1}^{R_{q}}6\mu_{\mathrm{jq}}-3\mu_{\mathrm{jq}}^{2}}}∼N\left(0,1\right),$$

where $\hat{k}=\left(\sum_{q}s_{S_{q}}^{2}+\sum_{q}s_{D_{q}}^{2}\right)/\left(3\Sigma_{j,q}\mu_{\mathrm{jq}}-\frac{19}{2}\Sigma_{j,q}\mu_{\mathrm{jq}}^{2}\right)$, obtained by similar reasoning (the notation $\Sigma_{j,q}$ is shorthand for the double summation in the previous formula). Note that $s_{S_{q}}^{2}$ and $s_{D_{q}}^{2}$ are computed as in the single region case, using all $S_{\mathrm{qi}}\;\,and\;D_{\mathrm{qi}},i=1,\text{…},N$ from region q. From this, the empirical version can be obtained similarly as above,

$$T_{\mathrm{ap}-\mathrm{emp}}=\frac{2\bar{D^{\pi }}-\bar{S^{\pi }}}{\sqrt{\frac{1}{N}\left(\sum_{q}s_{S_{q}}^{2}+\sum_{q}s_{D_{q}}^{2}\right)\left(2+\frac{4Q_{∙∙}}{3N}\right)}}∼N\left(0,1\right).$$

For the weighted test, a multiple region statistic can be obtained by adding the contributions across regions as well as across families. Keeping in mind that the observed IBD sharing of a sibpair can change from one region to the next, the derivation is similar to the single region test, leading to the expression

$$\begin{array}{ll} T_{\mathrm{ap}-w}= & \frac{1}{\sqrt{\sum_{q}n_{Z_{\mathrm{qi}}=1}+n_{Z_{\mathrm{qi}}=2}}}\sum_{q=1}^{p}T_{\mathrm{ap}-w}^{q}\sqrt{n_{Z_{\mathrm{qi}}=1}+n_{Z_{\mathrm{qi}}=2}}\\ & ∼N\left(0,1\right), \end{array}$$

where the $\hat{k}$ used for computing $T_{\mathrm{ap}-w}^{q},q=1,\text{…},p,$ is the one given immediately above.

We note that there is no pathway extension for Epstein's test; however, a simple approximation can be constructed by regressing allele counts on IBD state—we call this the “regression test”—and it can be easily extended to test a pathway. See Appendix A for details.

### 2.5 Robustness of allelic parity statistics to linkage and LD

Under the null hypothesis of no RV association within a region, we expect evidence for excess IBD sharing in the region to be unusual, although it is possible that excess sharing might be observed when the test region is close enough to be in linkage with a common variant susceptibility locus, but far enough away that the RVs are not in LD with it. All three test statistics use the *k* parameter to account for within‐region LD. In Appendix B, we show that linkage can inflate the allelic parity comparison but the bias will be negligible unless the set of RVs is exceptionally large. We conclude that $T_{\mathrm{ap}}$ and $T_{\mathrm{ap}-\mathrm{emp}}$ are reasonably robust to linkage, but as a precaution, we recommend that IBD sharing estimates be examined for regions suspected to harbor susceptibility genes. On the other hand, the $T_{\mathrm{ap}-w}$ statistic derives from conditional means and variances of $D_{i}$ given $Z_{i}$, so does not depend on IBD sharing values and thus is fully robust to the presence of linkage. This advantage, however, may be countered by lack of relevance or imprecision of the external population frequency $\mu_{j}$ values that can introduce bias into $T_{\mathrm{ap}-w}$.

In a series of simulation studies reported in the next section, we compare validity and power of the affected sibpair RV test statistics of interest under various design parameters, and investigate robustness of $T_{\mathrm{ap}-w}$ to MAF misspecification. Because all test statistics based on observed allele counts may be adversely affected by sequencing errors or the occurrence of de novo mutations, we also investigate robustness of methods to these practical issues. Finally, we evaluate the consequences of defining sets of RV with less rare MAF.

## 3 SIMULATION STUDIES

### 3.1 Design

Starting with 594 European haplotypes from the 1000 Genomes Project, we simulate a genetic region to be tested for association; the region, of length 13.6 kb, is taken arbitrarily from chromosome 1. Because the minimum MAF that can be simulated using the samples from the 1000 Genomes European haplotypes is 1/594 = 0.17%, to generate variants that are more rare, we first filter variants on MAF < 0.2%, and then add a sufficient number of “noncarrier” families (i.e., families with haplotypes containing the wild type variant at all of the rare loci) to bring the MAF of the entire pool of parental haplotypes below the desired threshold of 0.1% for all variants. We generate families of parents with two offspring using R package “sim1000G,” which assumes random haplotype pairing, random mating, and Mendelian inheritance (Dimitromanolakis, Xu, Krol, & Briollais, 2019).

Under the *alternative* hypothesis of RV association we generate age at onset for each individual offspring via a proportional hazards model with rate

(1) $$h\left(t|X\right)=h_{0}\left(t-t_{0}\right)\text{exp}\left(\Sigma_{j=1}^{R}\beta_{j}X_{j}\right),$$

where *h*_0_(*t*) is the baseline hazard function, which we specify as Weibull, and *t*_0_ is a minimum age of disease onset set to age 20. ***X*** is the individual‐level genotype vector indicating carrier (1) or noncarrier (0) of the rare allele at each of the *R* rare loci. Among these, there are *C* susceptibility loci, where *C* represents 15% of all RVs in the region, chosen at random. The parameters *β*_*j*_, *j* = 1, …, *R* correspond to effect sizes for RVs in the region (so that *β*_*j*_ > 0 for all of the *C* susceptibility variants, and *β*_*j*_ = 0 for the *R* − *C* nonrisk variants). We draw a family from the population pool of size 500,000 families, and apply the PH model (1) to generate the age at onset for each of the siblings. The model (1) is implemented in R package “FamEvent” (Choi, Kopciuk, He, & Briollais, 2017), and returns the cumulative distribution function (cdf) of the age at onset for one individual. The onset age is simulated for each of the siblings independently as the inverse cdf computed at a uniform random variate, under the individual PH model. We define “affected” as disease onset before age 50, and ascertain a pair into the study if both siblings are affected. The procedure of drawing from the pool and ascertaining is repeated until the target sample size *N* is obtained. The distribution of observed rare allele counts per sib pair in a single region, which is heavily weighted toward counts of zero, becomes visibly heavier in the right tail following ascertainment under the alternative (Figures S1 and S2).

Under the *null* scenario of no association, genotypes are simulated before ascertainment using “sim1000G” as detailed above, but none of the RVs are designated to be susceptibility loci in the region; effectively $\beta_{j}=0,$ for all j=1,…,R. This null is region‐specific, not global; in practical application, affected families without any susceptibility alleles in the region could be environmental, or genetic cases arising at some other region. To improve computational efficiency for the intensive null simulations, we do not generate age at onset for the offspring, but instead automatically ascertain families into the study. This is correct because phenotype is independent of genotype under the null hypothesis of no RV association in the region.

Under a pathway scenario, we generate RV genotypes in two genetically independent regions $G_{1}$ and $G_{2}$ on chromosomes 1 and 3, which are assumed to form a functional pathway. Extending the single region approach, there are $C_{1}$ and $C_{2}$ different risk variants in each region, representing 15% of RVs in each region, respectively. Their joint effect is captured through the function g(∙) in the genetic model: $h\left(t|X^{1},X^{2}\right)=h_{0}\left(t-t_{0}\right)\text{exp}\left(g\left(X^{1},X^{2}\right)\right)$. Under the null hypothesis, there is no RV association in either region. Under the alternative, we consider two different genetic architectures. In an additive model suggested in P. I. Lin, Vance, Pericak‐Vance, and Martin (2007), the effects of deleterious alleles are added across regions, although it is rare for one family to carry more than one such RV. In the epistatic model of Marchini, Donnelly, and Cardon (2005), rare susceptibility alleles are required at both genes for loss of function to occur (in this case, one gene acts as a “modifier” to the other). Since this scenario occurs rarely, we are more permissive with the MAF filtering to obtain a visible effect.

To compare performance of the association tests, we apply them in each data set generated under a null or an alternative genetic model; replicated datasets are drawn independently under single‐region and multiple region mechanisms, for combinations of four study sizes *N* (20, 100, 500, and 1,000 families), and various effect sizes (Table 3). Going forward we drop the original version of the allelic parity test and only include the empirical and weighted versions. We found the original version to have similar performance characteristics to the empirical version, but the additional requirement to specify allele frequencies, as well as lower power compared to the weighted version makes its use less appealing.

**Table 3:** Genetic models used to generate ascertained datasets in the power simulations

Model	Description	Simulation settings	Population MAF
Single region	$g=\sum_{j=1}^{R}\beta_{j}X_{j}$	$\beta_{j}=\log\left(\mathrm{HR}\right)$, for HR values of 2, 4, and 8.	<0.001
Multiple region, Additive model	$g=\sum_{j=1}^{R_{1}}\beta_{j1}X_{j}^{1}+\sum_{j=1}^{R_{2}}\beta_{j2}X_{j}^{2}$	$\beta_{j1}=\beta_{j2}=\log\left(\mathrm{HR}\right)$, for HR values of 2, 4, and 8.	<0.001
Multiple region, Epistatic model	$g=\beta \times I\left\{\sum_{j=1}^{R_{1}}X_{j}^{1}>0\right\}\times I\left\{\sum_{j=1}^{R_{2}}X_{j}^{2}>0\right\}$	β=log⁡(8)	<0.005

### 3.2 Validity and power

**Figure 1:**
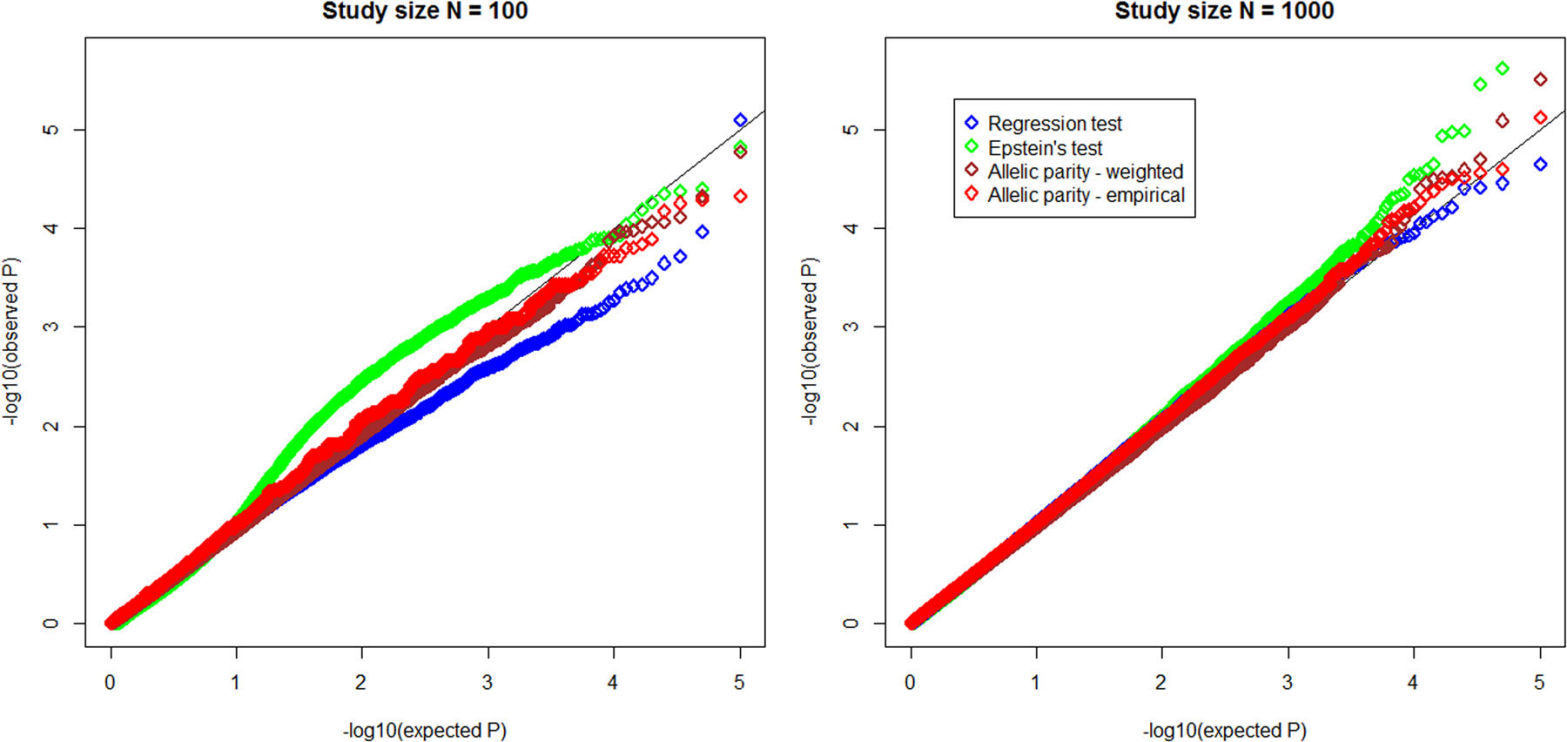
Q–Q plots of single‐region test statistic p‐values under the null hypothesis for sample sizes *N* = 100 and 1,000 families and 100,000 replicated data sets

**Figure 2:**
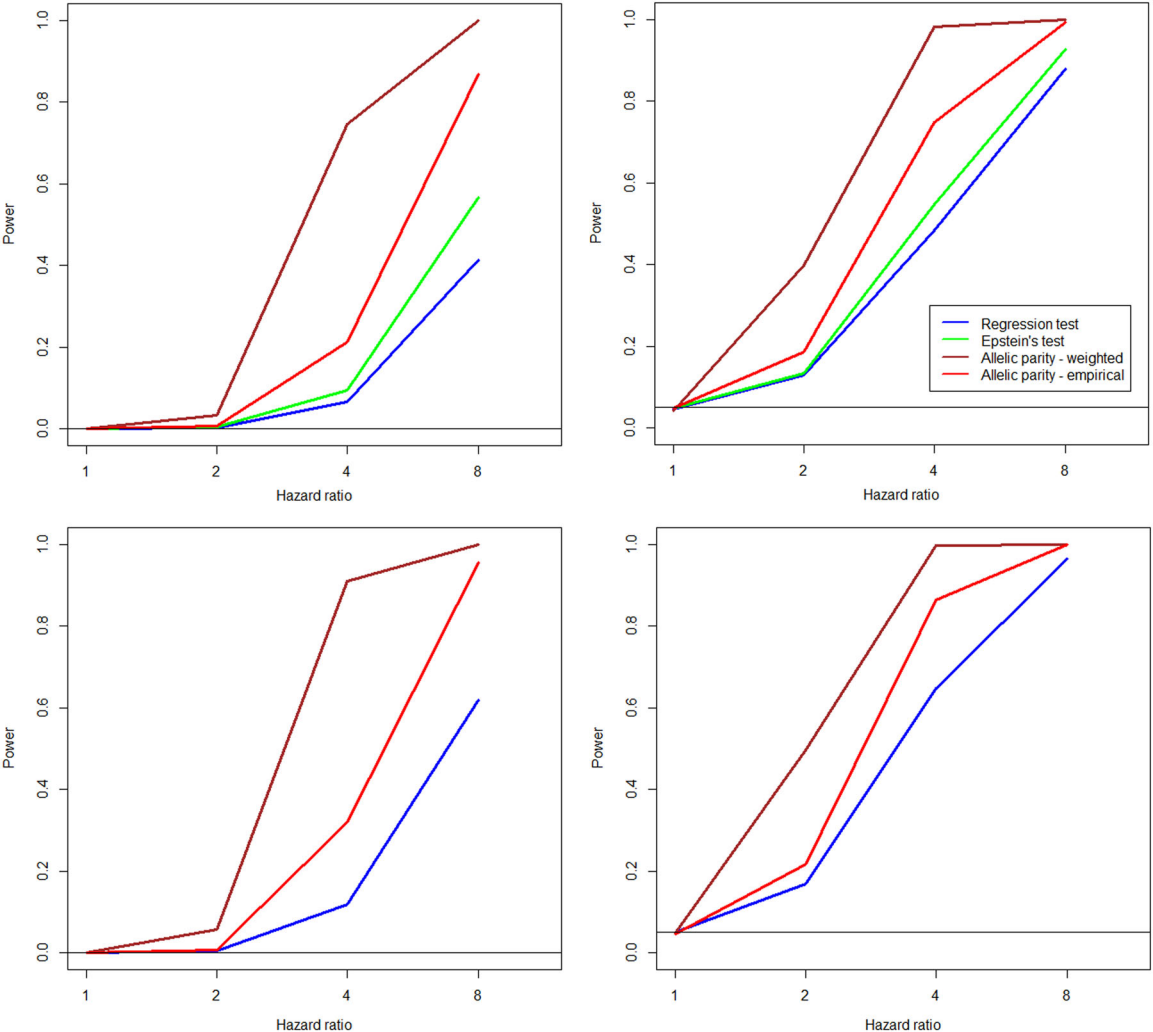
Power curves for testing at *α* = .0005 (left) and *α* = .05 (right) for a sample size *N* = 500, and 10,000 replicated datasets. Results for single region (top panels) and two‐region pathway under the additive model (bottom panels). The horizontal black lines represent the significance threshold *α*

**Figure 3:**
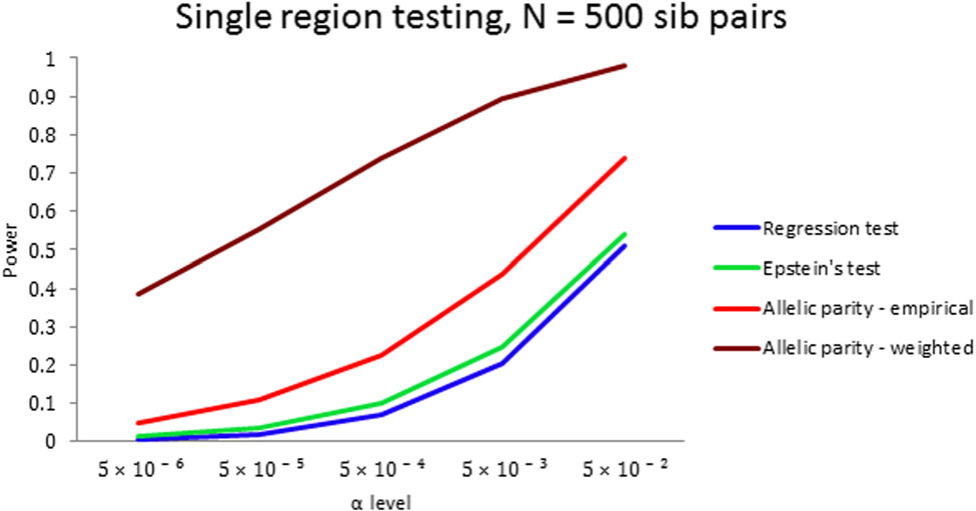
Power of single region testing versus significance threshold for medium penetrance variants (HR = 4) for sample size *N* = 500 sib pairs, and 100,000 replicated data sets

To assess type I error control, the observed *p* values (−log 10 transformed) for each test are plotted in Figure 1 versus those expected under the null, for *N* = 100 and 1,000, using 100,000 replicates. We see that the test size is well controlled. Plots for *N* = 500 show similar behavior; for *N* = 20 the empirical allelic parity test is conservative in the tail, however, the weighted version works well (Figure S3). For power calculations, we employ 10,000 replications, and estimate power as the fraction of tests that reject the null at level α, for data sets generated under the alternative models specified in Table 3. Power curves for a sample size of 500, evaluated at significance criteria α=.05 and .0005 (Figure 2) show that the allelic parity test—especially the weighted version—is more powerful by a factor of 2–10 compared to regression‐based tests. A more dramatic display of power differentials occurs at stricter significance levels (Figure 3), where the ratio increases with decreasing α. We observe that test rankings according to power do not depend on sample size (Figure S5).

Results for two‐region pathway testing are similar to single‐region testing under the null and additive models (Figures S4 and S6). Unsurprisingly, the power is higher in general for pathway testing compared to region testing (Figure 2). In particular, it is encouraging to see that a pathway with highly penetrant variants (HR = 8) can be detected in a sample as small as 20 sib pairs, with power just above 50% (Figure S6). Under the epistasis model, power is generally low, as expected. Still, the weighted allelic parity test performs visibly better than the other tests (Figure S7).

Finally, all results shown in the text refer to one‐sided tests. This is sensible at the genome‐wide level when testing single regions for deleterious variants. It is also possible to perform two‐sided tests, if we have reason to believe that in certain regions, RVs could be primarily protective.

### 3.3 Robustness

We perform additional simulation studies to evaluate practical consequences of misspecification of reference population parameters, sequencing errors and de novo mutations, as well as sensitivity to rare variant criteria.

(1) Misspecified external MAF estimates (the $\mu_{j}$'s) in the weighted allelic parity test. These evaluations generate random errors for the reference population MAFs, with random $\mu_{j}$'s drawn independently from an exponential distribution under three scenarios. The exponential mean is taken to be, in turn, underestimated (by a factor of 2) compared to the true MAF used in the prior simulations, equal, or over‐estimated (by a factor of 2). As might be expected, the test is liberal for under‐estimated MAFs and conservative for over‐estimated MAFs (Figure S8 and Table S1). For unbiased MAFs, type I error is well controlled for sample size *N* = 100, but becomes liberal with larger *N*.
  We suggest two relatively simple remedies to deal with misspecified $\mu_{j}$'s. The first is to use an adjusted null distribution, which is similar to the concept of an “empirical null” distribution from Efron (2004) (See Supporting Information Methods for details). Application of this strategy to the simulated misspecified test statistics yields an obvious improvement in performance. Type I errors become well controlled using the empirical null approach in the under‐estimated and unbiased scenarios, and only slightly conservative for over‐estimated $\mu_{j}$, and power estimates also become close in all three scenarios (Figure S8 and Table S1). An alternative remedy is to scale the $\mu_{j}$ estimates by a common factor so that they become unbiased in distribution. This approach is suitable when an empirical null distribution may not be available, such as when performing only one or a few tests. We show an example of using a scaling factor in the Application section.
(2) Sequencing errors in next generation sequencing and de novo mutations. Sequencing errors can cause base substitutions, which will appear as rare variants in the data used for analysis, and induce inaccuracies in RV tests if the error rate is high enough. We simulate sequencing errors and add these extra “rare alleles” to the genetic data, for prespecified error rates of 10%, 25%, and 50% among the observed rare variants. As a quality control step, we flag as errors and remove all alleles that contradict the sharing state for that particular locus and sib pair; this is only possible for an IBD state of 2 and an odd valued $Q_{\mathrm{ij}}$. De novo variants similarly add rare alleles that are not part of Mendelian inheritance. The same simulation setup is applicable to de novo mutations, except that, because they are quite rare (perhaps only 30 per genome), they have a less material impact. For all tests, Type I error and power (Table S2) decrease with increasing error rate. The empirical allelic parity test is the most affected by errors and the weighted test is least affected. This is sensible, since errors will inflate the $S_{i}$ counts of single alleles, whereas the $D_{i}$'s would require an error to occur at the site of an pre‐existing rare allele, which is less likely. We note that even though all tests become conservative, the weighted allelic parity test retains high power (above 95%) even for an error rate of 50%. We recommend this test for use in the presence of sequencing errors. We also note that bioinformatics tools may be able to weed out common sequencing errors; for instance, Ma et al. (2019) report an approach that can dramatically reduce the A > T substitution error rate in deep sequencing data.
(3) Comparative test performance for low frequency variants. The development of our methods was motivated by the aim of uncovering very rare variants (MAF < 0.1%), but the tests can be applied with more common variants as well (e.g., low frequency variants). Type I error simulations for MAF < 3% and MAF < 5% show that, compared to the regression tests which retain close to nominal type I error control, the allelic parity tests are conservative, but not extremely so. All tests tend to have higher power for low frequency than for rare variants, as expected under a simplistic simulation model, where the HR is constant at all MAFs (Table S3). The allelic parity tests have the highest power, with the weighted version being the most powerful. It may be possible to improve type I error control for the weighted version by including higher order terms ($O\left(\mu_{j}^{3}\right)$ and higher) in the expressions for mean and variance of $D_{i}$; this investigation is reserved for future work.

### 3.4 Software and code

A function that implements the tests with an example data set, as well as the code files used in the simulation studies are included as Supporting Information Material. The function is for general use, and can be run in R. The simulation code is intended only for the purpose of replicating the results in this paper, and runs in a multicore Unix environment.

## 4 APPLICATION: AFFECTED SISTER PAIRS WITH EARLY‐ONSET BREAST CANCER (BC)

A woman's risk of developing BC increases with the number of close family members diagnosed (Collaborative Group on Hormonal Factors in Breast Cancer, 2001; O'Brien et al., 2016). This risk is even higher if a family member is diagnosed at a young age (before 45 years). However, known genes with variants predisposing individuals to hereditary BC explain less than 50% of disease clustering within families (Easton et al., 2015; A. Lee et al., 2019). The motivating data set is a pilot study of whole exome sequencing (WES) in ASPs with a family history of cancer and early‐onset in at least one sibling. The median age at diagnosis is 45 years, and all but one family have one sib diagnosed before age 45. The ASPs, recruited from the Ontario Familial Breast Cancer Registry (John et al., 2004; Terry et al., 2015), had been screened negative for known mutations in susceptibility genes (including *BRCA1/2* and CHEK2*1100delC variants), thereby increasing the chances of finding rare familial mutations; all families except for one were classified as Caucasian. The pilot data set included 37 individuals from 17 families (14 pairs and three triplets). We count triplets as three pairs, yielding N=23 observations at the ASP level.

In total, 251,931 variants were annotated with MAF information obtained from three reference panels: 1000 Genomes Project (n=1,092; all populations), Exome Sequencing Project (n=6,500), and UK Biobank (n=500,000). Variants were deemed rare if they appeared in at least one of the panels (using the entire populations for improved precision), and if the maximum MAF from these references was no greater than 0.5%. As a QC step, rare variant loci that were missing in more than a few (4) families were excluded, otherwise missing genotypes were imputed to be the rare allele. Other standard QC procedures followed Genome Analysis Toolkit Best Practice recommendations, and included haplotype calling, variant recalibration, conversion to human genome version hg19, annotation by ANNOVAR, and filtering on read depth and quality. This resulted in 18,035 rare variants that passed quality control, annotated to 9,572 genes (the number of RVs per gene ranged from 1 to 69, with mean 1.9).

We specified the population parameters $\mu_{j}$, used in the weighted allelic parity test, as the median MAF at locus j across the three reference panels. However, we observed that the samples were enriched in rare variants across the exome, in comparison to the allele frequencies in the panels. Therefore, we applied a simple multiplicative genome‐wide adjustment factor of 10.1 chosen to match the panel frequencies cumulated at the gene level to the observed frequencies, (see Figure S8 for the details of the calculation). This rescaling amounts to converting an over or underestimated scenario to an unbiased one, which is closest to nominal performance, as per the simulations in Section 3.3, part 1. We note that we used the entire UK Biobank data which includes RVs imputed from genotype data, and that a similar enrichment in exome RVs was found in an exome sequenced subset of the UK Biobank, compared with the entire panel MAFs (imputed from genotype data). Van Hout et al. (2019) report a >fourfold increase in coding variants, and >10‐fold increase in loss‐of‐function variants identified in WES compared with imputed data, with rare variants accounting for the vast majority of this increase.

To determine the IBD sharing in each sib pair, we analyzed 102,322 common autosomal variants (MAF > 0.10) using the multipoint algorithm implemented in MERLIN (Abecasis, Cherny, Cookson, & Cardon, 2001). Sex‐averaged linkage map positions were downloaded from Rutgers University's Map Interpolator. IBD estimates were obtained on genomic segments (“clusters”) defined adaptively so that *R* ^2^ among any two SNPs in a cluster is more than 0.1 (Abecasis & Wigginton, 2005; Abecasis, n.d.); this improves stability and accuracy of IBD sharing estimates in the absence of parental data. Finally, pairwise IBD sharing estimates for ASPs in each family were obtained on 6,899 clusters spanning chromosomes 1–22.

To illustrate single gene and pathway testing, we aimed to validate a known BC‐related functional pathway—DNA repair. If successful, this might help identify previously unreported variants within this pathway as potential hereditary mutations. Pathway information was taken from Dexheimer (2013) and includes 84 genes known to be involved in the various mechanisms of molecular DNA repair; 41 of these genes had at least one RV, hence could be tested. Table 4 reports seven genes with the top *p* values for the weighted allelic parity tests. This test has the smallest *p* values among the tests considered, and the top two genes, *BLM* and *MLH1*, reach significance accounting for multiple testing (at level 0.05/41=0.0012). For pathway level analysis, we first tested the whole DNA repair pathway, and then we tested its component pathways, each having a different biological role in DNA repair (Table 5). The *p* value for testing the entire DNA repair pathway (significant at the 5% level) is smaller than the *p* values for each of the component sub‐pathways; it is also smaller than the *p* value of the top gene (*MLH1*), suggesting aggregate testing can be effective. This confirms our intuition that the signal is dispersed throughout the pathway, and shows that multiple region testing can provide information not captured with gene‐level testing.

**Table 4:** Top hits for genes in DNA repair pathways (*p* value (ap‐w) < 0.1), rows are ordered by *p* value of the allelic parity‐weighted test

Gene	Chrom	*R* ^a^	*p*‐val_Regression_	*p*‐val_Epstein_	*p*‐val_a.p. empirical_	*p*‐val_a.p. weighted_	Pathway^b^
MLH1	3	5	0.08	0.04	0.001	0.0002	2
BLM	15	3	0.35	0.13	0.003	0.0009	4
ERCC4	16	1	0.31	0.14	0.009	0.0014	3
XPC	3	3	0.18	0.29	0.047	0.0076	3
POLL	10	2	0.24	0.11	0.047	0.0082	5
POLD3	11	1	0.32	–	0.085	0.022	1, 3
XRCC3	14	1	0.50	0.45	0.085	0.030	4

*Note*: Full results are given in Table S1.

^a^ *R* is the number of RV loci in the gene.

^b^ Pathway codes are: 1, base excision repair; 2, mismatch repair; 3, nucleotide excision repair; 4, homologous recombination; and 5, nonhomologous end‐joining.

**Table 5:** Pathway testing of DNA repair mechanisms (separately and jointly)

Pathway	*R* ^a^	*T*_Regression_	*p*‐val_Regression_	*T*_a.p. empirical_	*p*‐val_a.p. empirical_	*T*_a.p. weighted_	*p*‐val_a.p. weighted_
Base excision repair	26	−1.14	0.87	0.81	0.21	1.35	0.09
Mismatch repair	16	0.90	0.19	1.54	0.06	0.97	0.17
Nucleotide excision repair	20	1.58	0.07	1.19	0.12	3.46	2.7E−04
Homologous recombination	26	−0.31	0.62	−0.42	0.66	0.39	0.35
Nonhomologous end‐joining	17	0.21	0.42	0.94	0.17	2.02	0.02
DNA repair (all mechanisms)	83	0.43	0.34	0.83	0.20	3.67	1.2E−04

^a^ *R* is the number of RV loci in the pathway.

## 5 DISCUSSION

In this communication, we consider the problem of discovery of rare variants in a sample of ASPs. Our methodological findings make headway in two directions: first, we develop powerful testing methods for this particular study design at the region level. Second, we extend these methods for use at the genetic pathway level. The allelic parity test is novel, to our knowledge, and offers important advantages compared to the other methods considered. It has good type I error properties, and the weighted version can be more powerful than the other tests as evident in all simulation scenarios considered. The power advantage comes at the price of sensitivity to the accuracy of the external RV frequency values, but we propose that this can be remediated by use of an empirical null distribution method. Moreover, we find good robustness to sequencing errors and de novo mutations, as well as to rare variant criteria.

The performance of the allelic parity methods over tests that regress allele count on IBD state can be explained by the fact that allele parity counting (whether alleles appear as singles or duplicates) is a better discriminator between susceptibility and null regions at the sib pair level, compared to IBD state. This is illustrated graphically in Figure S2 in the Supporting Information, which plots allele enrichment under the null and alternative. The regression of counts versus IBD goes from a slope of zero (under the null) to a positive slope (under the alternative), and this is captured by the regression tests. However, a simple linear regression cannot capture the fact that, when IBD is 1, the ratio of duplicate alleles to single copies (i.e., ${2D}_{i}:S_{i}$) also increases (>1), which is extra information used by the allelic parity test. Also notable is the general enrichment in rare alleles for all IBD sharing states. This is missed by all tests except for the weighted allelic parity, which compares counts against a baseline level, supplied externally.

We expect that the allelic parity tests we propose will be robust to confounding by population structure or environmental factors, with some caveats. The empirical test compares double and single allele counts within each sibpair and sums up this difference, which is strictly a within‐family comparison and therefore robust to population stratification. For environmental exposures shared by the sibpair, the empirical version will be similarly robust. However, a need remains for evaluation of extensions that can account for individual‐specific risk factors such as age at menarche. For the weighted version which incorporates a population comparison, robustness to population stratification requires that the external allele frequencies accurately reflect the population structure of the sample families. This means that frequencies should be obtained for each population group, after which a pooled $\mu_{j}$ estimate would be computed with weights chosen to match the genetic diversity represented in the sample. As larger more accurate reference population panels are becoming available, it is increasingly feasible to closely match samples to their background population MAFs. With this setup, the denominators in $T_{\mathrm{ap}-w}$ could be expressed as aggregate differences within ancestry groups, provided that the same variance in the denominators can be used across groups. With a large enough sample, one could relax this assumption and attempt to standardize the $D_{i}$'s using different variance estimates for different ancestry groups. The weighted version would likely not be robust to other confounders, but it may be possible to incorporate relevant covariates into this and other test statistics, and further work to investigate such extensions is warranted.

Testing at the pathway level can be informative, especially when small to moderate effects are distributed across functional pathways, a setting in which it would be impossible to detect association at the single region level without a very large sample. Because testing at the pathway level will inevitably include a large number of null variants in the statistics, the signal in a pathway should be rich enough overall, and distributed broadly enough for the tests to be successful at detecting it. Besides power, the other benefit of pathway testing is that it can offer functional insight into the etiology of disease, beyond what a single gene might indicate. Once a pathway has been identified and validated, it follows naturally to examine each component gene (or RV) separately, to gain a deeper understanding of how the pathway operates as a network.

Beyond methodological improvements, implications for study design deserve to be brought to the forefront. Previous authors have reported that the affected sibling design is more cost effective than case/control studies (Epstein et al., 2015; K. H. Lin & Zöllner, 2015; Zöllner, 2012). In particular, for single gene testing, Epstein et al. (2015) demonstrate a twofold power gain for sib pair testing (500 pairs) compared to case–control comparisons (500 each), on average over different effect sizes, and assuming that shared environmental and other genetic factors between sibs do not have a very strong effect on diagnosis. It stands to reason then, since our best test is routinely 2–10 times more powerful than Epstein's, that even under conservative scenarios applying it with an ASP design is likely to compound the power gains compared with case–control. A practical limitation of the ASP design is the availability of ASPs for sequencing. However, at least for studies in which the barrier is cost of sequencing rather than availability of subjects, the proposed test should be of significant interest to investigators looking to detect novel rare variants.

## Supporting information

Supporting information

Supporting information

Supporting information

Supporting information

## Data Availability

Summary data that support the findings of this study are available on request from the senior author. Individual data are not publicly available due to privacy or ethical restrictions.

## APPENDIX A EPSTEIN'S TEST AND THE REGRESSION TEST

### Epstein's test

Briefly, the method of Epstein et al. (2015) involves testing for the slope of the regression of $Q_{i∙}$ on $Z_{i}$ being positive, namely $H_{0}:\mu_{1}-\mu_{0}=0$, as seen from the main text (recall that *Q*_*i*._ = Σ_*j*=1, …, *R*_ *Q*_*ij*_). This is accomplished by first centering the two variables as $\tilde{Q_{i∙}}=Q_{i∙}-\sum_{i=1}^{N}{W_{i}Q}_{i∙}$ and $\tilde{Z_{i}}=Z_{i}-\sum_{i=1}^{N}{W_{i}Z}_{i}$, where $W_{i}$ is an estimate of ${\left(\mathrm{Var}\left(Q_{i∙}|Z_{i}\right)\right)}^{-1}$. The authors show that an efficient score to test $H_{0}$ is proportional to $U=\sum_{i=1}^{N}W_{i}\tilde{Q_{i∙}}\tilde{Z_{i}}$, which has an estimated variance $V=\sum_{i=1}^{N}{\left\{W_{i}\tilde{Q_{i∙}}\tilde{Z_{i}}\right\}}^{2}-N{\left(U/N\right)}^{2}$. Hence their $Y_{\mathrm{burden}}=U/\sqrt{V}∼N\left(0,1\right)$.

The quantities $W_{i}$ require estimates of variance parameters $\sigma_{0}^{2}\;\,and\;\,\sigma_{1}^{2}$. These are calculated as $\left(\begin{array}{c} {\hat{\sigma }}_{0}^{2}\\ {\hat{\sigma }}_{1}^{2} \end{array}\right)={\left(X^{T}X\right)}^{-1}X^{T}{\left({\hat{V}}_{0},{\hat{V}}_{1},{\hat{V}}_{2}\right)}^{T}$, where $X=\left[\begin{array}{cc} 4 & 0\\ 2 & 4\\ 0 & 8 \end{array}\right]$, and ${\hat{V}}_{0}$, ${\hat{V}}_{1}$, and ${\hat{V}}_{2}$ are the sample variances for the counts of rare alleles possessed by affected sib pairs (ASPs) sharing 0, 1, or 2 alleles IBD, and are computed directly from data.

### Regression test

We also propose a simpler version of Epstein's test that assumes only one variance parameter (instead of two). This corresponds more closely to standard regression, and is preferable when data are insufficient to estimate sample variances ${\hat{V}}_{0}$, ${\hat{V}}_{1}$, and ${\hat{V}}_{2}$, for example, when N is small, and MAF is low. For a single (contiguous) region, consider the regression

$$Q_{i∙}=\alpha +\beta Z_{i}+\epsilon_{i},\epsilon_{i}∼N\left(0,\sigma^{2}\right),\;\text{for}\;\text{all}\;i=1,\text{…},N$$

which is implemented via the *lm*() function in R. The test of β=0 versus *H*_a_: β≠0 has the form

$$T_{\mathrm{reg}}=\frac{\hat{\beta }}{\mathrm{SE}\left(\hat{\beta }\right)}∼t\left(N-2\right).$$

At the pathway level, let $Q_{\mathrm{qi}∙}=\Sigma_{j=1,\text{…},R_{q}}Q_{\mathrm{qij}}$, for all regions q=1,2,…,p, and the regression equation is

$$\begin{array}{l} Q_{\mathrm{qi}∙}=\alpha +\beta Z_{\mathrm{qi}}+\epsilon_{i},\;\epsilon_{\mathrm{qi}}∼N\left(0,\sigma^{2}\right),\;\\ \text{for}\;\text{all}\;i=1,\text{…},N\;\text{and}\;q=1,\text{…},p. \end{array}$$

The test statistic for β=0 versus β≠0 is asymptotically normal

$$T_{\mathrm{reg}}=\frac{\hat{\beta }}{\mathrm{SE}\left(\hat{\beta }\right)}∼N\left(0,1\right).$$

## APPENDIX B DERIVATION OF NULL MEANS AND VARIANCES OF ALLELE COUNTS

To compute the expected values of $S_{i}$ and $D_{i}$, we first derive the conditional probabilities $P\left(Q_{\mathrm{ij}}|Z_{i}\right)$ for all the combinations of $Q_{\mathrm{ij}}=1,2$ and $Z_{i}=0,1,2$, where $\mu_{j}$ is the mean number of rare alleles observed at locus j on a single haplotype in the general population.

$$\begin{array}{l} P\left(Q_{\mathrm{ij}}=1|Z_{i}=0\right)=P\;(\text{rare}\;\text{allele}\;\text{at}\;\text{locus}\;j\\ \;\text{on}\;\text{one}\;\text{of}\;4\;\text{haplotypes})=4\mu_{j}{\left(1-\mu_{j}\right)}^{3}\\ =4\mu_{j}-12\mu_{j}^{2}+12\mu_{j}^{3}-4\mu_{j}^{4}, \end{array}$$

$$\begin{array}{l} P\left(Q_{\mathrm{ij}}=1|Z_{i}=1\right)\\ \;=P(\text{only}\;\text{rare}\;\text{allele}\;\text{on}\;\text{one}\;\text{of}\;2\;\text{non}‐\text{shared}\\ \;\text{haplotypes})=2\mu_{j}\left(1-\mu_{j}\right)\left(1-\mu_{j}\right)\\ \;=2\mu_{j}-4\mu_{j}^{2}+2\mu_{j}^{3}, \end{array}$$

$$\;P\left(Q_{\mathrm{ij}}=1|Z_{i}=2\right)=0,$$

$$\begin{array}{l} P\left(Q_{\mathrm{ij}}=2|Z_{i}\;=0\right)\\ \;=P(\text{rare}\;\text{all}\text{ele}\;\text{at}\;\text{locus}\;j\;\text{on}\;\text{exactly}\;2\;\\ \;\text{of}\;4\;\text{haplotypes})=\left(\begin{array}{c} 4\\ 2 \end{array}\right){\mu_{j}^{2}\left(1-\mu_{j}\right))}^{2}\\ \;=6\mu_{j}^{2}-12\mu_{j}^{3}+6\mu_{j}^{4}, \end{array}$$

$$\begin{array}{l} P\left(Q_{\mathrm{ij}}=2|Z_{i}=1\right)\\ \;=P(\text{rare allele on both non}‐\text{shared haplotypes}\;\,\\ \;and\;\text{none on shared haplotypes})\\ \;+P(\text{rare allele on shared haplotypes}\;and\;\,\\ \;\text{none on others})\\ \;=\mu_{j}^{2}\left(1-\mu_{j}\right)+\mu_{j}{\left(1-\mu_{j}\right)}^{2}=\mu_{j}-\mu_{j}^{2}, \end{array}$$

$$\begin{array}{l} P\left(Q_{\mathrm{ij}}=2|Z_{i}=2\right)\\ \;=P(\text{rare alleles on one pair of shared haplotypes}\\ \;and\;\text{none on the other pair})\\ \;=2\mu_{j}\left(1-\mu_{j}\right)=2\mu_{j}-2\mu_{j}^{2}, \end{array}$$

Then, $E\left(D_{i}\right)$ and $E(S_{i})$ are computed by the law of total expectation where $f_{0}=P\left(Z_{i}=0\right)$, with $f_{1}$ and $f_{2}$ defined similarly

$$\begin{array}{l} E\left(D_{i}\right)=\sum_{j=1,\text{…},R}E\left(I\left\{Q_{ij}=2\right\}\right)=\sum_{j=1,\text{…},R}P\left(Q_{ij}=2\right)\\ \;=\sum_{j=1,\text{…},R}P\left(Q_{ij}=2|Z_{i}=0\right)P\left(Z_{i}=0\right)\\ \;+P\left(Q_{ij}=2|Z_{i}=1\right)P\left(Z_{i}=1\right)\\ \;+P\left(Q_{ij}=2|Z_{i}=2\right)P\left(Z_{i}=2\right)\\ \;=\sum_{j=1,\text{…},R}\left(6\mu_{j}^{2}-12\mu_{j}^{3}+6\mu_{j}^{4}\right)f_{0}+\left(\mu_{j}-\mu_{j}^{2}\right)f_{1}\\ \;+\left(2\mu_{j}-2\mu_{j}^{2}\right)f_{2}\\ \;=\sum_{j=1,\text{…},R}\mu_{j}\left(f_{1}+2f_{2}\right)+\mu_{j}^{2}\left(6f_{0}-\left(f_{1}+2f_{2}\right)\right)\\ \;+O\left(\mu_{j}^{3}\right)\\ E\left(S_{i}\right)=\sum_{j=1,\text{…},R}2\mu_{j}\left(f_{1}+2f_{0}\right)-4\mu_{j}^{2}\left(f_{1}+3f_{0}\right)+O(\mu_{j}^{3}), \end{array}$$

and

$$\begin{array}{l} E\left(2D_{i}-S_{i}\right)=\sum_{j=1,\text{…},R}4\mu_{j}\left(f_{2}-f_{0}\right)+24\mu_{j}^{2}f_{0}\\ \;+2\mu_{j}^{2}\left(f_{1}-2f_{2}\right)+O\left(\mu_{j}^{3}\right). \end{array}$$

In the absence of linkage, which we expect to be the usual case under the null of no RV association: we have $\left(f_{0},f_{1},f_{2}\right)=\left(\frac{1}{4},\frac{1}{2},\frac{1}{4}\right),\left(f_{1}+2f_{0}\right)=1,\left(f_{2}-f_{0}\right)=0,\left(f_{1}-2f_{2}\right)=0$. Thus $E\left(D_{i}\right)$ simplifies to $E\left(D_{i}\right)=\sum_{j=1,\text{…},R}\mu_{j}+\frac{1}{2}\mu_{j}^{2}-3\mu_{j}^{3}+\frac{3}{2}\mu_{j}^{4}$ = $\sum_{j=1,\text{…},R}\mu_{j}+\frac{1}{2}\mu_{j}^{2}+O\left(\mu_{j}^{3}\right)$, and $E\left(S_{i}\right)=\sum_{j=1,\text{…},R}2\mu_{j}-5\mu_{j}^{2}+4\mu_{j}^{3}-\mu_{j}^{4}=\sum_{j=1,\text{…},R}2\mu_{j}-5\mu_{j}^{2}+O\left(\mu_{j}^{3}\right)$, similarly.

Therefore, under the null hypothesis

$$\begin{array}{l} E\left(2\bar{D}-\bar{S}\right)=\sum_{j=1,\text{…},R}6\mu_{j}^{2}-10\mu_{j}^{3}+4\mu_{j}^{4}\\ \;=\sum_{j=1,\text{…},R}6\mu_{j}^{2}+O\left(\mu_{j}^{3}\right). \end{array}$$

In the unusual case of linkage under the null (i.e., excess sharing in the region), the first order bias $(4\left(f_{2}-f_{0}\right)\sum_{j=1,\text{…},R}\mu_{j})$ could be greater than zero when $f_{2}>f_{0}$. In the case of rare variants with MAF < 0.005, the sum of population allele frequencies in the region will be modest unless the set of RVs is quite large. Calculations of bias in the numerator $2\overset{®}{D}-\overset{®}{S}$ for 41 DNA pathway genes (Table S4) in the WES data (not shown) suggest that bias under the null is small (specifically, in absolute value, the bias is on average 3.3% of the absolute value of the numerator).

To calculate the variance of $S_{i}$ and $D_{i}$, we make the assumption $Var(D_{i})=k\sum_{j=1,\text{…},R}Var\left(I\left\{Q_{ij}=2\right\}\right),$ where the parameter k captures the effect of LD between loci. We generally expect correlation to be small between rare loci, however, we cannot assume independence (k=1) since this would lead to inaccuracies. Variance at a single locus j can be computed as

$$\begin{array}{l} Var\left(I\left\{Q_{ij}=1\right\}\right)=E\left[I^{2}\left\{Q_{ij}=1\right\}\right]-{\left[E\left(I\left\{Q_{ij}=1\right\}\right)\right]}^{2}\\ \;=P\left(Q_{ij}=1\right)-{\left(P\left(Q_{ij}=1\right)\right)}^{2}\\ \;=2\mu_{j}-9\mu_{j}^{2}+O\left(\mu_{j}^{3}\right). \end{array}$$

The variance further simplifies to $Var\left(D_{i}\right)=k\sum_{j=1,\text{…},R}\mu_{j}-\frac{1}{2}\mu_{j}^{2}+O\left(\mu_{j}^{3}\right)$, and similarly $Var\left(S_{i}\right)=k\sum_{j=1,\text{…},R}2\mu_{j}-9\mu_{j}^{2}+O\left(\mu_{j}^{3}\right)$. The conditional variances $\mathrm{Var}\left(D_{i}|Z_{i}=1,2\right)$, used as weights in the allelic parity weighted test, are derived following the same process. To estimate k we use the sample variances of $S_{i}$ and $D_{i}$ computed from data ($s_{S}^{2}$ and $s_{D}^{2}$), and combine them to obtain

$$\hat{k}=\frac{s_{S}^{2}+s_{D}^{2}}{\Sigma_{j=1,\text{…},R}3\mu_{j}-\frac{19}{2}\mu_{j}^{2}}.$$

This factor which is defined as the ratio of $\mathrm{Var}(D_{i})$ to the sum of variances of its individual terms accounts for linkage disequilibrium (LD) within a region being tested for RV association. The idea is to let the data inform *k*. When there is no LD, then *k* should be estimated to be close to 1, but in the presence of LD, *k* will be >1 due to positive correlation between RVs, and hence produces a higher $\mathrm{Var}(D_{i})$ compared to the summed variances (over *j*) of $I\left\{Q_{\mathrm{ij}}=2\right\}$.

## APPENDIX C DERIVATION OF THE ALLELIC PARITY TEST STATISTIC

Derivation for the variance of $2\overset{®}{D}-\overset{®}{S}$:

$$\begin{array}{l} Var\left(2I\left\{Q_{ij}=2\right\}-I\left\{Q_{ij}=1\right\}\right)=4Var\left(I\left\{Q_{ij}=2\right\}\right)\\ \;+Var\left(I\left\{Q_{ij}=1\right\}\right)\\ \;-4Cov\left(I\left\{Q_{ij}=1\right\},I\left\{Q_{ij}=2\right\}\right)\\ \;=6\mu_{j}-3\mu_{j}^{2}+O(\mu_{j}^{3}), \end{array}$$

where we use the fact that $Cov\left(I\left\{Q_{ij}=1\right\},I\left\{Q_{ij}=2\right\}\right)=-2\mu_{j}^{2}+O\left(\mu_{j}^{3}\right)$. It follows easily then that $Var\left(2\bar{D}-\bar{S}\right)=\frac{1}{N}k\sum_{j=1,\text{…},R}6\mu_{j}-3\mu_{j}^{2}+O\left(\mu_{j}^{3}\right)$.

Derivation for empirical version of $T_{\mathrm{ap}-\mathrm{emp}}$:

We make use of the result: $\frac{{\Sigma_{j}a}_{1}\mu_{j}+a_{2}\mu_{j}^{2}+O\left(\mu_{j}^{3}\right)}{\Sigma_{j}b_{1}\mu_{j}+b_{2}\mu_{j}^{2}+O\left(\mu_{j}^{3}\right)}=\frac{a_{1}}{b_{1}}+\frac{a_{2}b_{1}-a_{1}b_{2}}{b_{1}^{2}}\Sigma_{j}\mu_{j}+O\left(\mu_{j}^{2}\right)$.

The denominator of $T_{\mathrm{ap}}$, under the square root, is

$$\begin{array}{l} \frac{1}{N}\hat{k}\left(6\sum_{j}\mu_{j}-3\sum_{j}\mu_{j}^{2}\right)=\frac{1}{N}\left(s_{S}^{2}+s_{D}^{2}\right)\frac{\Sigma_{j}6\mu_{j}-3\mu_{j}^{2}}{\Sigma_{j}3\mu_{j}-\frac{19}{2}\mu_{j}^{2}}\\ \;=\frac{1}{N}\left(s_{S}^{2}+s_{D}^{2}\right)\left(\frac{6}{3}+\frac{-3\cdot 3+6\cdot \frac{19}{2}}{3^{2}}\sum_{j}\mu_{j}+O\left(\mu_{j}^{2}\right)\right)\\ \;=\frac{1}{N}\left(s_{S}^{2}+s_{D}^{2}\right)\left(2+\frac{16}{3}\Sigma_{j}\mu_{j}+O\left(\mu_{j}^{2}\right)\right), \end{array}$$

via the previous result. Ignoring the second order power of the $\mu_{j}$'s and estimating Σ_*j*_ *μ*_*j*_ by $Q_{∙∙}/4N$ leads us to the formula in the text.
