# Supplementary material for: Gene‐based and pathway‐based testing for rare‐variant association in affected sib pairs
Source: Genet Epidemiol. 2020 Apr 1;44(4):368–81. doi: 10.1002/gepi.22291 (PMC7318298; doi:10.1002/gepi.22291)
Supplement: Supplementary file 1 — Supporting information [file GEPI-44-368-s001.docx]

**Supporting Information**

**Figure S1**. Histograms of observed RV frequency counts in a sample size of 10,000 sibpairs ascertained under the null (top panels) and alternative (bottom panels). Left side panels count all RVs, right side panels only risk RVs. Frequencies are given on a log scale.


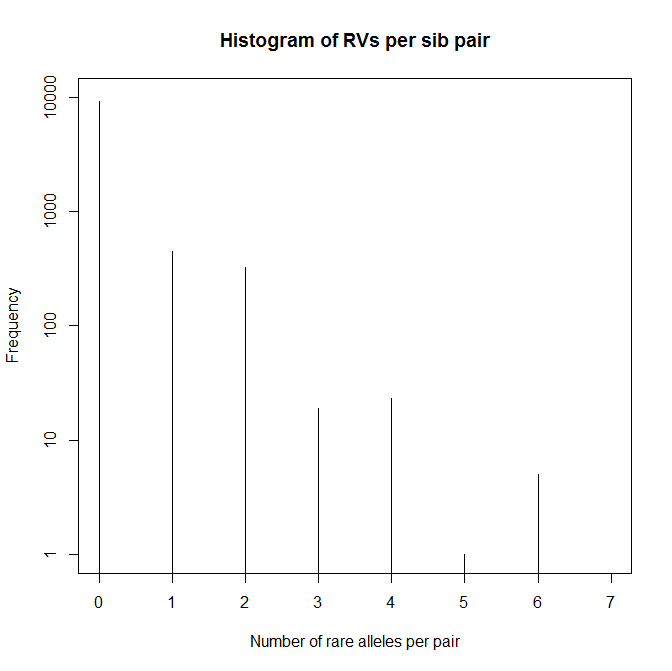

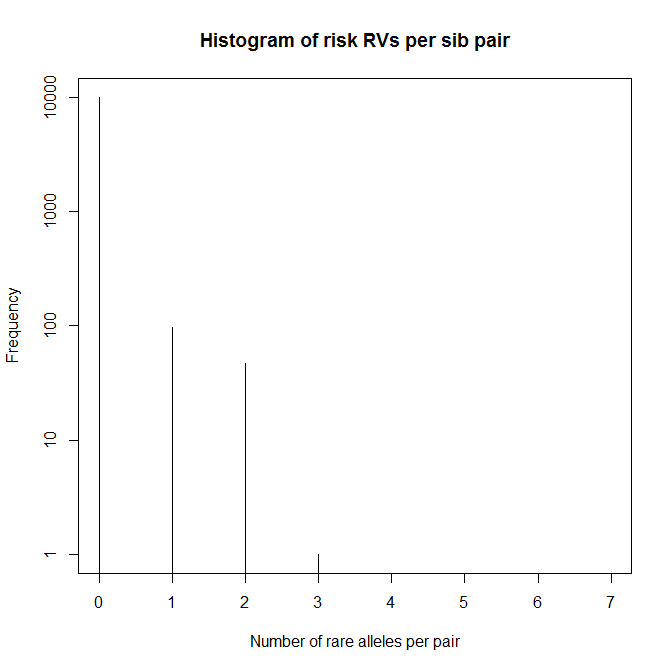


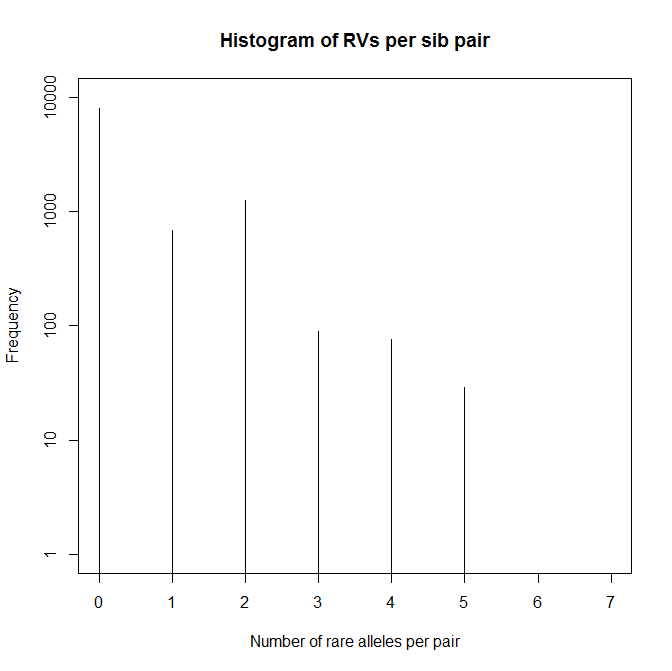

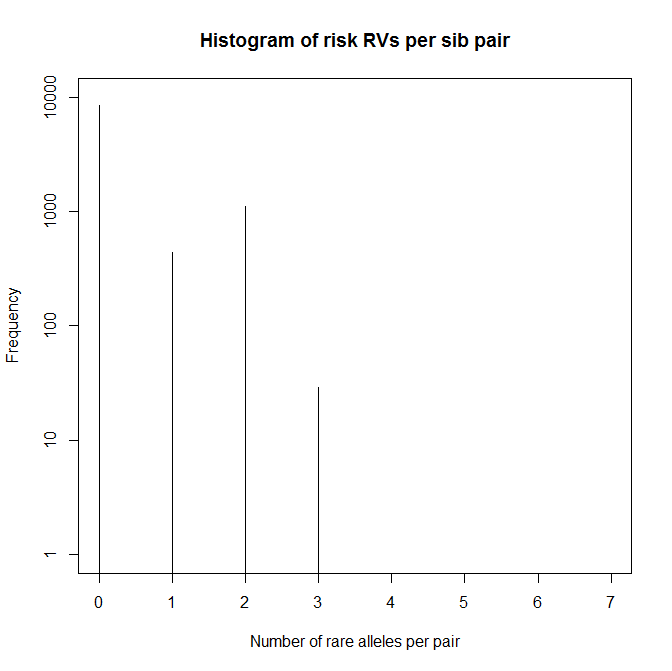


**Figure S2**. Average observed counts of single and duplicate alleles - under the null (left) and under the alternative (right) in an ascertained sample size of 10,000 sibpairs, stratified by IBD sharing state.

**Figure S3**. Q-Q plots of single-region test statistic p-values (-log10) under the null hypothesis for sample sizes N = 20, 100, 500 and 1000 sibpairs and 100,000 replicated datasets.


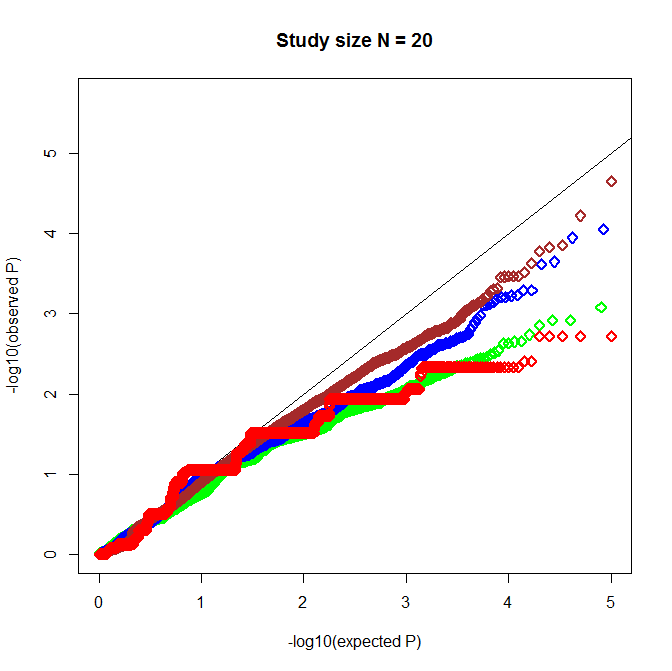

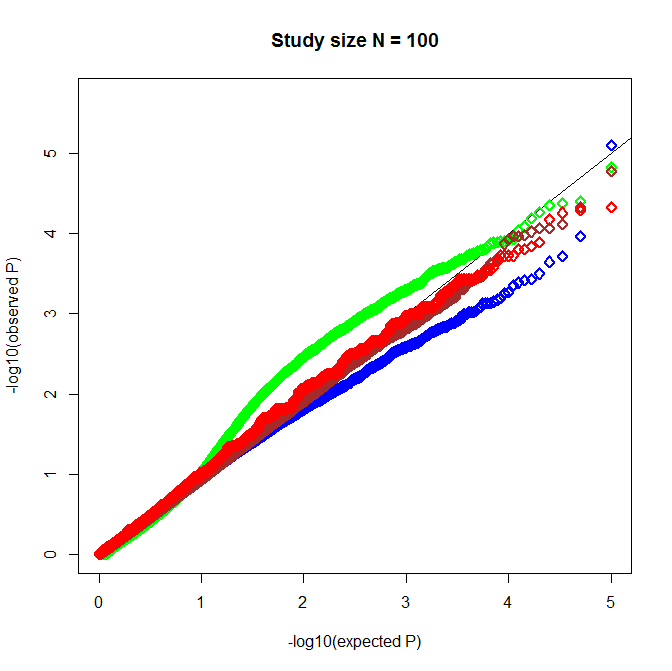


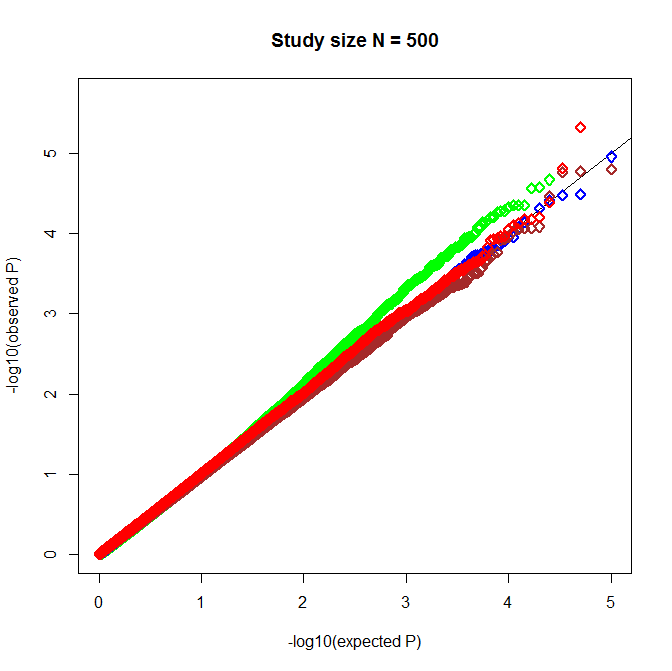

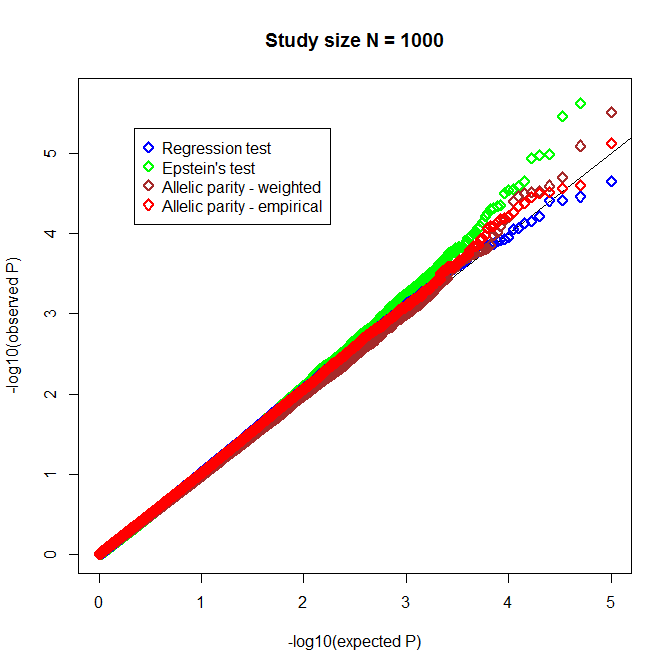


**Figure S4**. Q-Q plots of two-region pathway test statistic p-values (-log10) under the null hypothesis for sample sizes N = 20, 100, 500 and 1000 sibpairs and 100,000 replicated datasets.


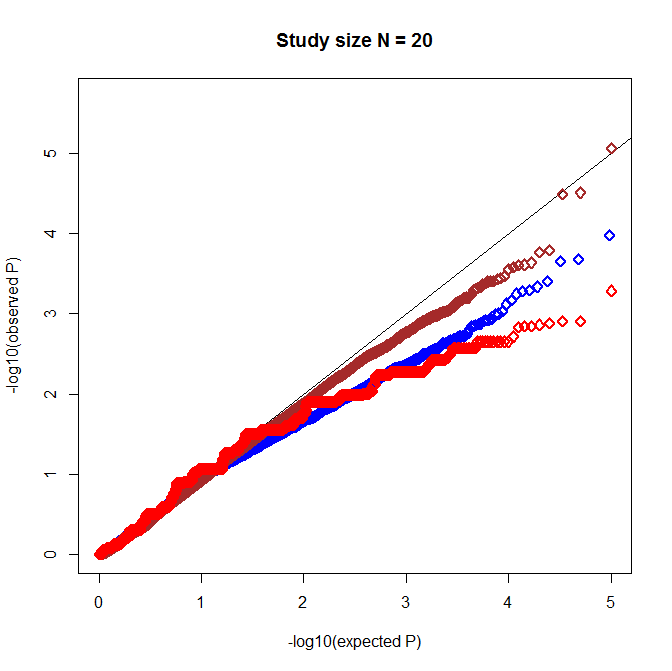

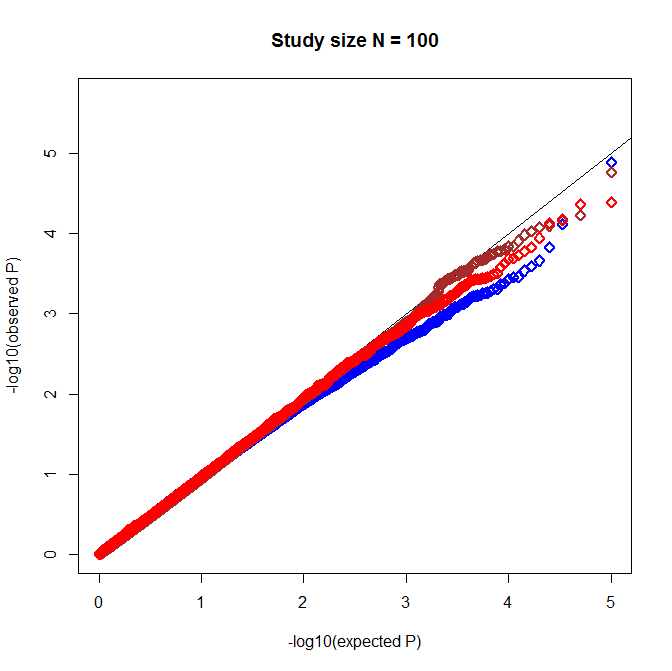

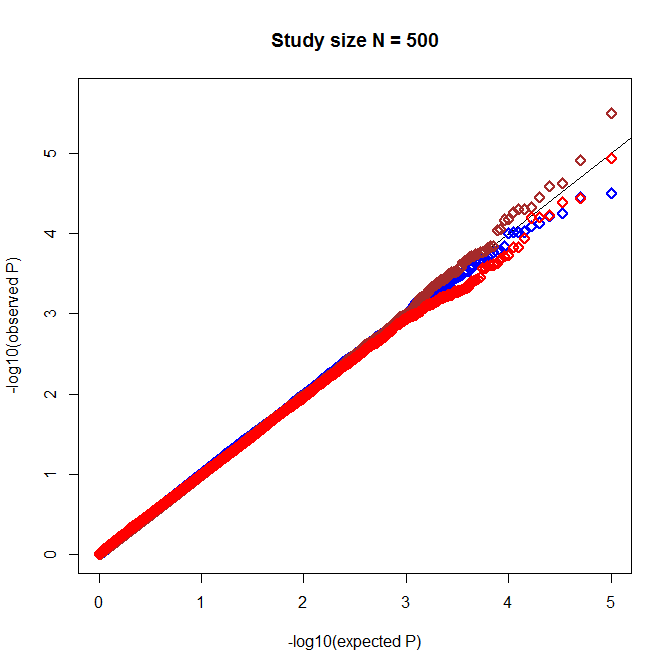

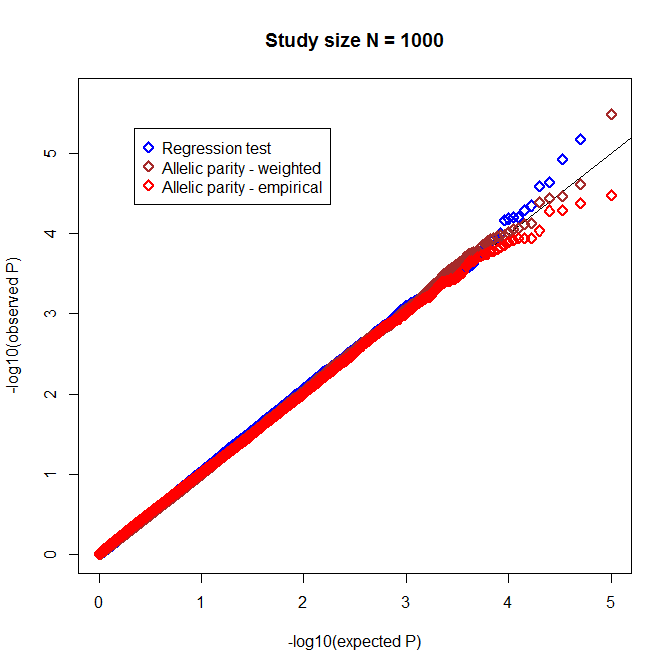


**Figure S5.** Power for single region testing for various sample sizes, from 10,000 replicated datasets. Effect size is HR = 8, and significance levels are 0.0005 (top), and 0.05 (bottom).

**Figure S6**. Power for two region additive pathway testing for various sample sizes, from 10,000 replicated datasets. Effect size is HR = 8 and significance levels 0.0005 (top), and 0.05 (bottom).

**Figure S7.** Power for two region pathway testing, under the epistatic model, for various sample sizes, from 10,000 replicated datasets. Effect size is HR = 8.

**Robustness Studies**

*1. Misspecification of allele frequencies*.

We simulate misspecified external allele frequencies for the single gene region discussed in the text under each of three scenarios:

i) unbiased MAFs, where allele frequencies $\mu_{j}, j=1,\ldots,R$ are i.i.d. Exponential with mean equal to the true frequency used in the simulated (null) population;

ii) under-specified, with allele frequencies generated from an Exponential with mean one half the true frequency; and

iii) over-specified, in which case the Exponential has a mean of double the true frequency. Once generated for each replicated dataset, the misspecified MAFs are used to calculate the allelic parity test statistic (weighted version).

We also consider adjusting the null distribution as a way to make the analysis robust to misspecified external MAF estimates. This adjustment, which we will refer to as the ‘empirical null distribution’ (not to be confused with the empirical version of the allelic parity test) consists of looking at the distribution of the test statistics ($T_{ap-w})$ obtained from running a full GWAS, and comparing it to the theoretical null distribution. One should expect that all genes, with potentially a few exceptions, fall under the null hypothesis. Thus, if the theoretical distribution of the test statistics does not match the observed, then a parameterized version of the observed (i.e., empirical) distribution is used to compute p-values instead. Specifically for $T_{ap-w}$ we suggest using a normal with a mean set to the median of the observed statistics, and a variance matching the sample variance of the same. The concept of an ‘empirical null’ was introduced in Efron (2004) as a way to correct p-values for model misspecification.

Figure S8 shows the type I error results under all three misspecification scenarios, using both the theoretical and empirical null distributions. Power results are given in Table S1 for $HR=4$ and significance level 0.05.

**Table S1.** Power of single-region tests under misspecified external MAFs, for various sample sizes, from 10,000 replicated datasets.

|  |  | Using theoretical p-values | | | Using empirical null p-values | | |
| --- | --- | --- | --- | --- | --- | --- | --- |
|  | N = | 100 | 500 | 1000 | 100 | 500 | 1000 |
| Bias | 0.5 × | 0.790 | 1.000 | 1.000 | 0.570 | 0.967 | 0.998 |
| (× true) | 1 × | 0.567 | 0.975 | 0.998 | 0.557 | 0.961 | 0.994 |
|  | 2 × | 0.188 | 0.436 | 0.564 | 0.461 | 0.919 | 0.969 |
| Exact MAFS | | 0.555 | 0.982 | 1.000 |  |  |  |

**Figure S8.** Type I errors for single-region testing under misspecification of external MAFs: under-estimated (top), unbiased (middle) and over-estimated (bottom). Left panels use the theoretical null to compute p-values, right panels use the empirical null. The three curves are for sample sizes N=100, 500, and 1000 sibpairs, from 100,000 replicated datasets.


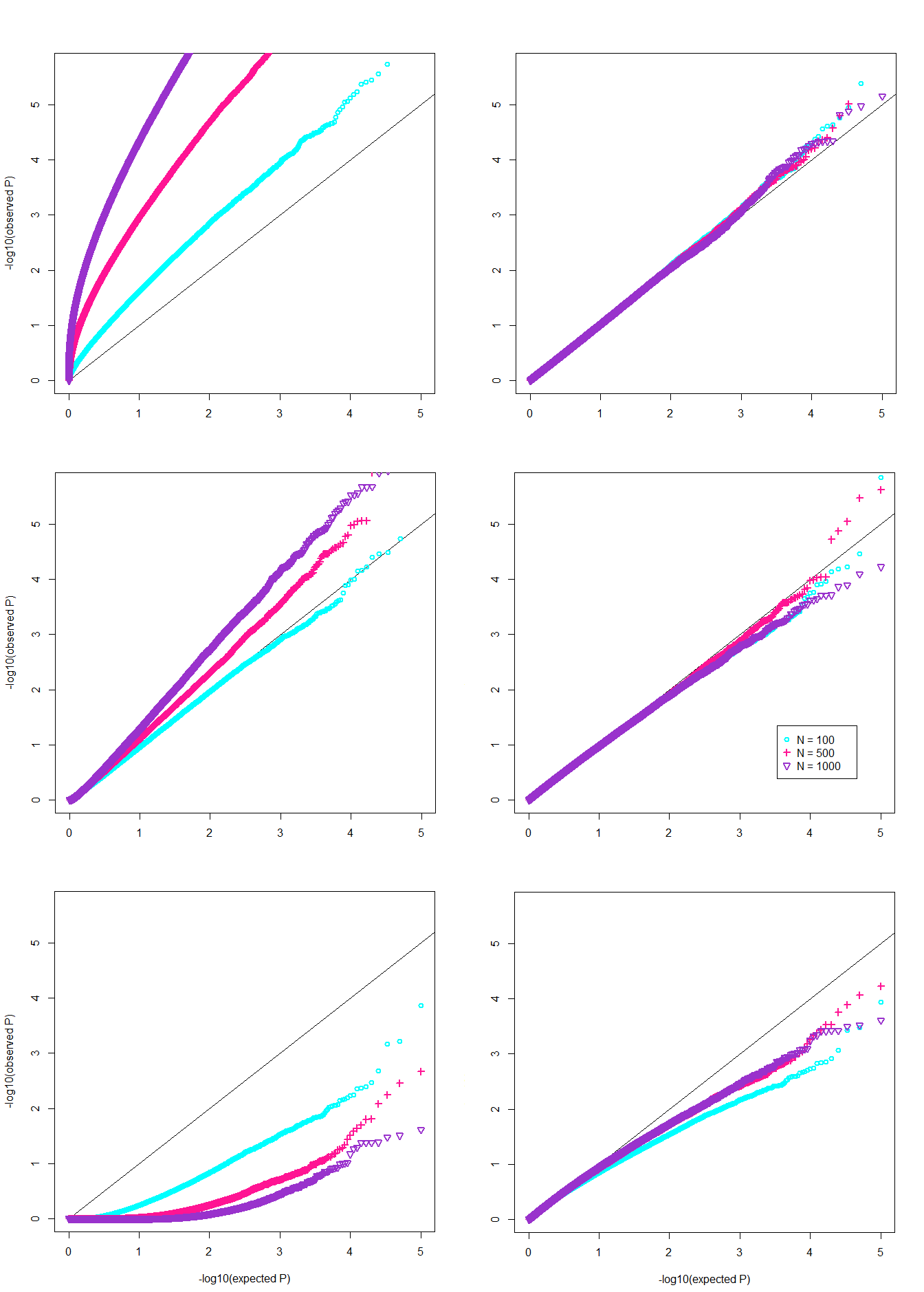


*2. Sensitivity to sequencing errors.* We investigate the impact on performance of the various tests under the assumption that the genetic data we observe are subject to error, at a specific rate ($x \%$), expressed as a fraction of all observed rare alleles. To simulate the errors, we first simulate the genotype as in the paper, after which we add a random error at each genetic locus $Q_{ij}$. The errors come from i.i.d. Poisson distributions with mean $4\mu_{j} x/(100-x)$. This ensures that $x \%$ of all observed rare variation is due to errors in sequencing. Tests are performed in ignorance of which rare alleles are errors, with the only correction being to exclude genotypes inconsistent with the sharing state, e.g., if $Q_{ij}=1$ at a locus with $Z_{ij}=2$, then the rare allele is dropped as an error, and $Q_{ij}$ is set to 0 for the analysis. We perform type I error and power simulations for a sample size $N=500$ sibpairs and a hazard ratio of 4, using 10,000 replications, and a significance level of 0.05. Table S2 shows results assuming error rates of 10, 25 and 50%. Values in the absence of error (0%) are included for comparison.

**Table S2.** Type I error and power of single-region tests under simulated sequencing errors, from 10,000 replicated datasets. The error rate gives the percentage of rare alleles that are called as such due to sequencing errors.

| Error rate (%) |  |  | Type I |  |  |  | Power |  |
| --- | --- | --- | --- | --- | --- | --- | --- | --- |
|  | 0 | 10 | 25 | 50 | 0 | 10 | 25 | 50 |
| Regression test | 0.049 | 0.027 | 0.008 | 0.001 | 0.5107 | 0.426 | 0.312 | 0.086 |
| Epstein’s test | 0.052 | 0.032 | 0.014 | 0.002 | 0.5418 | 0.473 | 0.374 | 0.147 |
| Allelic parity – empirical | 0.052 | 0.015 | 0.001 | 0 | 0.7383 | 0.608 | 0.36 | 0.031 |
| Allelic parity – weighted | 0.046 | 0.036 | 0.027 | 0.014 | 0.9816 | 0.982 | 0.973 | 0.961 |

*3. Sensitivity to raising the MAF threshold.* This investigates how raising the maximum MAF threshold for rare variant set definition affects the performance of the methods. We perform type I error and power simulations for a sample size $N=500$ sibpairs and a hazard ratio of 1.5, using 10,000 replications, and a significance level of 0.05. Results in Table S3 assume upper thresholds for inclusion of 0.03 and 0.05.

**Table S3.** Type I error (alpha=0.05) and power of single-region tests that include low frequency variants. Criteria for inclusion of variants in the analysis are MAF < 0.03 or 0.05. Results are for HR = 1.5, using 10,000 replicated datasets.

|  |  | Type I |  | Power |
| --- | --- | --- | --- | --- |
| MAF | < 0.03 | < 0.05 | < 0.03 | < 0.05 |
| Regression test | 0.050 | 0.051 | 0.298 | 0.393 |
| Epstein’s test | 0.054 | 0.056 | 0.315 | 0.428 |
| Allelic parity – empirical | 0.030 | 0.025 | 0.342 | 0.446 |
| Allelic parity – weighted | 0.041 | 0.036 | 0.811 | 0.865 |

**Simulation details and R Code**

Simulation code is provided with the submission in file: Rcode.v6.submitted.txt,

to be run with Unix script file RJob_submit.sh, which executes the code in parallel on 50 nodes. This code implements the genetic simulation described in the text, and produces an output file that stores one replication per row, with columns being the various test statistics, for each of three study sizes.

An R function that implements the allelic parity test is given in the source file Rfunction.ap.txt, which takes as inputs the genetic data, sib pair definitions, and IBD sharing states (example templates provided with the submission), and returns the test statistic and p-value. Settings include the test version (weighted or empirical), and an option for one- or two-sided p-value.

**Figure S9.** Genome-wide adjustment to panel derived MAFs for WES data analysis. Observed cumulative frequencies at the gene level (for 9,572 genes), given by $Q_{\cdot\cdot}/4N$ where $Q_{\cdot\cdot}=\sum_{j=1,..,R;i=1,..,N} Q_{ij}$, are first compared with panel cumulative MAFs in a QQ plot (top left). This shows a linear pattern for most genes, contained in the red rectangle. Restricting the analysis to genes with $Q_{\cdot\cdot}\leq20$ and$\sum_{j} {MAF}_{j}<0.015$, which account for 98.7% of all genes, makes this match in distribution easier to see (top right). This suggests that a linear transformation is appropriate, and the adjustment factor is computed as the slope from regressing cumulated observed versus panel RV frequencies, without an intercept. Scatter plot including best fit line of $Q_{\cdot\cdot}/4N$ vs.$\sum_{j=1,\ldots,R} {MAF}_{j}$ panel, for the reduced gene set (bottom). The slope is 10.112, and the adjusted R-squared of the fit is 0.6078. Points are displayed slightly staggered on the y-axis to show the number of genes in each horizontal streak.


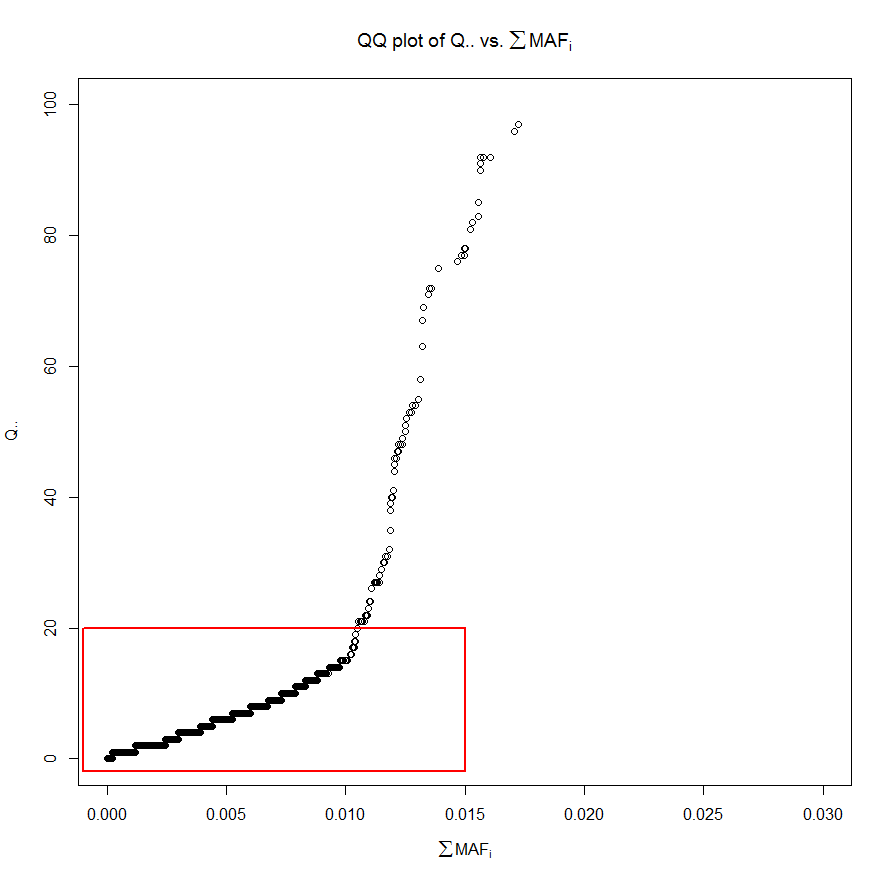

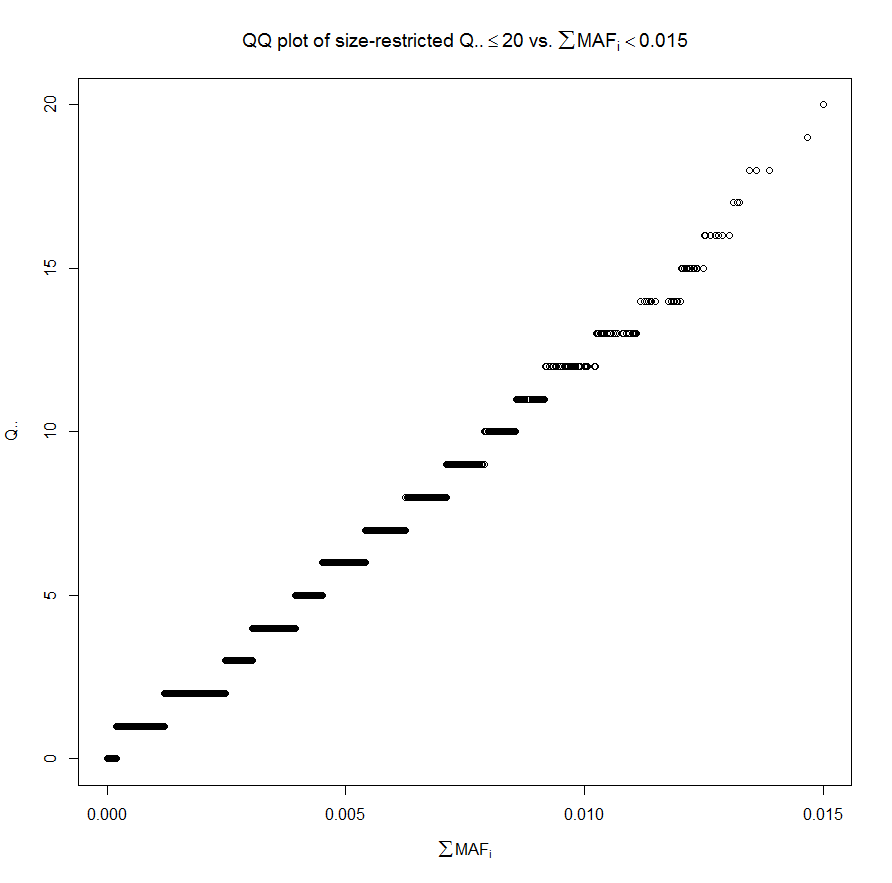


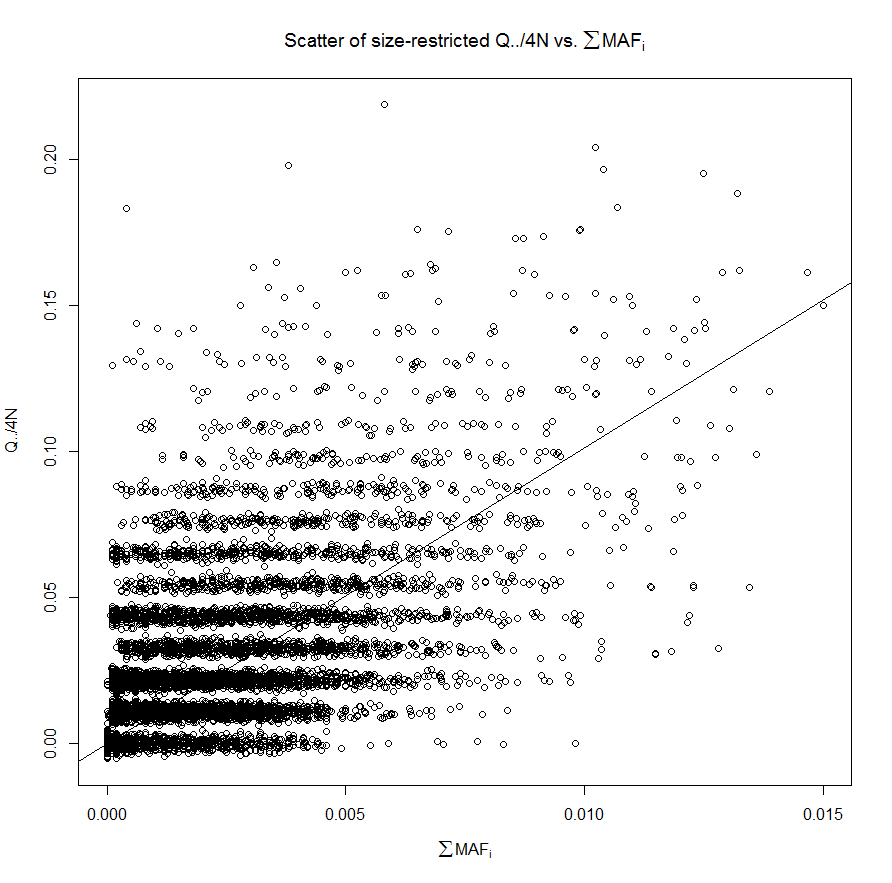


**Table S4.** Gene-based testing for the DNA repair pathway, ordered on p-value a.p. weighted. Results are shown only for genes which had at least 1 RV after filtering. Pathway codes are given in the main text.

| Gene | chr | R ^a^ | T_Regression_ | p-val _Regression_ | T_Epstein_ | p-val_Epstein_ | T_a.p. empirical_ | p-val_a.p. empirical_ | T_a.p. weighted_ | p-val_a.p. weighted_ | Pathway |
| --- | --- | --- | --- | --- | --- | --- | --- | --- | --- | --- | --- |
| *MLH1* | 3 | 5 | 1.49 | 0.08 | 1.70 | 0.04 | 3.08 | 0.0010 | 3.58 | *0.0002* | 2 |
| *BLM* | 15 | 3 | 0.40 | 0.35 | 1.11 | 0.13 | 2.74 | 0.0031 | 3.12 | *0.0009* | 4 |
| *ERCC4* | 16 | 1 | 0.49 | 0.31 | 1.07 | 0.14 | 2.37 | 0.0089 | 3.00 | 0.0014 | 3 |
| *XPC* | 3 | 3 | 0.92 | 0.18 | 0.55 | 0.29 | 1.67 | 0.047 | 2.43 | 0.0076 | 3 |
| *POLL* | 10 | 2 | 0.72 | 0.24 | 1.24 | 0.11 | 1.67 | 0.047 | 2.40 | 0.0082 | 5 |
| *POLD3* | 11 | 1 | 0.48 | 0.32 | – | – | 1.37 | 0.085 | 2.01 | 0.022 | 1,3 |
| *XRCC3* | 14 | 1 | 0.00 | 0.50 | 0.12 | 0.45 | 1.37 | 0.085 | 1.89 | 0.030 | 4 |
| *MSH6* | 2 | 1 | 0.07 | 0.47 | 0.34 | 0.37 | 1.37 | 0.085 | 1.25 | 0.105 | 2 |
| *XRCC1* | 19 | 6 | -0.75 | 0.77 | -2.24 | 0.99 | 0.67 | 0.252 | 1.16 | 0.123 | 1 |
| *RPRD1B* | 20 | 2 | 0.00 | 0.50 | -1.00 | 0.84 | 1.37 | 0.085 | 0.92 | 0.180 | 5 |
| *NEIL1* | 15 | 1 | -1.06 | 0.85 | -1.10 | 0.86 | 0.00 | 0.500 | 0.86 | 0.194 | 1 |
| *POLM* | 7 | 2 | -0.41 | 0.66 | -1.44 | 0.92 | -0.34 | 0.634 | 0.86 | 0.194 | 5 |
| *PMS2* | 7 | 1 | 1.32 | 0.10 | 0.15 | 0.44 | 1.37 | 0.085 | 0.70 | 0.242 | 2 |
| *PNKP* | 19 | 1 | 1.62 | 0.06 | 0.60 | 0.27 | 1.37 | 0.085 | 0.51 | 0.306 | 1,5 |
| *POLE* | 12 | 1 | 1.20 | 0.12 | 0.08 | 0.47 | 1.37 | 0.085 | 0.38 | 0.353 | 1,3 |
| *DDB1* | 11 | 1 | 1.58 | 0.06 | -0.69 | 0.75 | 1.37 | 0.085 | 0.31 | 0.377 | 3 |
| *WRN* | 8 | 3 | -0.35 | 0.63 | -1.40 | 0.92 | -0.34 | 0.634 | 0.20 | 0.420 | 5 |
| *APEX1* | 14 | 1 | -0.12 | 0.55 | -0.56 | 0.71 | 1.37 | 0.085 | 0.12 | 0.454 | 1,5 |
| *EXO1* | 1 | 5 | 0.20 | 0.42 | -0.50 | 0.69 | 0.22 | 0.414 | -0.06 | 0.523 | 2,4,5 |
| *MSH3* | 5 | 1 | -2.11 | 0.98 | -0.82 | 0.79 | -0.70 | 0.757 | -0.08 | 0.532 | 2 |
| *ERCC2* | 19 | 2 | -0.21 | 0.58 | -0.78 | 0.78 | -0.70 | 0.757 | -0.17 | 0.567 | 3 |
| *POLD2* | 7 | 3 | 0.38 | 0.35 | 0.98 | 0.16 | -0.70 | 0.757 | -0.17 | 0.568 | 1,3 |
| *RDM1* | 17 | 1 | -1.11 | 0.86 | 0.51 | 0.31 | -0.70 | 0.757 | -0.23 | 0.589 | 4 |
| *SMUG1* | 12 | 1 | -0.07 | 0.53 | -0.28 | 0.61 | -0.70 | 0.757 | -0.24 | 0.595 | 1 |
| *POLD1* | 19 | 4 | 1.08 | 0.15 | -0.51 | 0.70 | -0.34 | 0.634 | -0.31 | 0.623 | 1,3 |
| *RBBP8* | 18 | 2 | -1.06 | 0.85 | 1.23 | 0.11 | -1.00 | 0.840 | -0.33 | 0.631 | 4 |
| *RAD52* | 12 | 3 | -0.76 | 0.77 | – | – | -1.14 | 0.872 | -0.34 | 0.635 | 4 |
| *MSH2* | 2 | 1 | 0.07 | 0.47 | 0.34 | 0.37 | -0.70 | 0.757 | -0.38 | 0.649 | 2 |
| *NLRP2* | 19 | 4 | -1.27 | 0.89 | -1.77 | 0.96 | -0.62 | 0.732 | -0.55 | 0.709 | 4 |
| *XRCC5* | 2 | 1 | -1.21 | 0.88 | 0.73 | 0.23 | -0.70 | 0.757 | -0.58 | 0.720 | 5 |
| *NBN* | 8 | 2 | 0.29 | 0.39 | -0.71 | 0.76 | -1.23 | 0.891 | -0.59 | 0.721 | 4 |
| *OGG1* | 3 | 2 | -2.24 | 0.98 | -0.12 | 0.55 | -1.00 | 0.840 | -0.60 | 0.725 | 1 |
| *ERCC3* | 2 | 2 | -1.05 | 0.85 | -1.52 | 0.94 | -1.00 | 0.840 | -0.69 | 0.755 | 3 |
| *POLE3* | 9 | 1 | -0.76 | 0.77 | -1.50 | 0.93 | -1.00 | 0.840 | -0.71 | 0.762 | 1,3 |
| *MUS81* | 11 | 2 | 0.38 | 0.35 | 0.93 | 0.18 | -0.69 | 0.754 | -0.76 | 0.776 | 4 |
| *MSH4* | 1 | 1 | -0.36 | 0.64 | -0.92 | 0.82 | -0.70 | 0.757 | -0.77 | 0.779 | 2 |
| *MUTYH* | 1 | 3 | -4.17 | 1.00 | 1.55 | 0.06 | 1.67 | 0.047 | -0.88 | 0.811 | 1 |
| *RAD51B* | 14 | 3 | -0.96 | 0.82 | – | – | -1.00 | 0.840 | -1.10 | 0.865 | 4 |
| *POLB* | 8 | 1 | – | – | – | – | – | – | – | 1.000 | 3 |
| *MLH3* | 14 | 1 | – | – | – | – | – | – | – | 1.000 | 2 |
| *ERCC1* | 19 | 1 | – | – | – | – | – | – | – | 1.000 | 1 |

^a^ R is the number of RV loci in the pathway.
