# Supplementary material for: Gene‐based and pathway‐based testing for rare‐variant association in affected sib pairs
Source: Genet Epidemiol. 2020 Apr 1;44(4):368–81. doi: 10.1002/gepi.22291 (PMC7318298; doi:10.1002/gepi.22291)
Supplement: Supplementary file 4 — Supporting information [file GEPI-44-368-s004.rtf]

#!/bin/bash 
module unload mvapich2/2.2
module load openmpi3
module load R #R/3.5.0 

cd /home/your_account/GWAS


NSLOTS=50
mpirun -np $NSLOTS  -display-map -display-allocation  /home/your_account/R/x86_64-pc-linux-gnu-library/3.5/snow/RMPISNOW < /home/your_account/GWAS/Rcode.v6.submitted.txt

#And the sbatch command is 
#$ sbatch -p biostat -n 50 --mem-per-cpu=1000  ./RJob_submit.sh
